# MIIP downregulation drives colorectal cancer progression through inducing peri-cancerous adipose tissue browning

**DOI:** 10.1186/s13578-023-01179-0

**Published:** 2024-01-20

**Authors:** Qinhao Wang, Yuanyuan Su, Ruiqi Sun, Xin Xiong, Kai Guo, Mengying Wei, Guodong Yang, Yi Ru, Zhengxiang Zhang, Jing Li, Jing Zhang, Qing Qiao, Xia Li

**Affiliations:** 1 State Key Laboratory of Cancer Biology, Department of Biochemistry and Molecular Biology, Fourth Military Medical University, Xi’an, 710032 Shaanxi China; 2 Department of Pharmacology, Medical College, Yan’an University, Yan’an, 716000 Shaanxi China; 3 Key Laboratory of Resource Biology and Biotechnology in Western China, Ministry of Education, College of Life Sciences, Northwest University, Xi’an, 710069 Shaanxi China; 4 Department of Burns and Cutaneous Surgery, Xijing Hospital, Fourth Military Medical University, Xi’an, 710032 Shaanxi China; 5 Department of Orthopedics, Xijing Hospital, Fourth Military Medical University, Xi’an, 710032 Shaanxi China; 6 Key Laboratory of Resource Biology and Biotechnology in Western China, Ministry of Education. School of Medicine, Northwest University, Xi’an, 710069 Shaanxi China; 7 Department of General Surgery, Tangdu Hospital, Fourth Military Medical University, No. 569 Xinsi Road, Xi’an, 710038 Shaanxi China

**Keywords:** MIIP, Colorectal cancer, AZGP1, Adipose tissue browning

## Abstract

**Background:**

The enrichment of peri-cancerous adipose tissue is a distinctive feature of colorectal cancer (CRC), accelerating disease progression and worsening prognosis. The communication between tumor cells and adjacent adipocytes plays a crucial role in CRC advancement. However, the precise regulatory mechanisms are largely unknown. This study aims to explore the mechanism of migration and invasion inhibitory protein (MIIP) downregulation in the remodeling of tumor cell-adipocyte communication and its role in promoting CRC.

**Results:**

MIIP expression was found to be decreased in CRC tissues and closely associated with adjacent adipocyte browning. In an in vitro co-culture model, adipocytes treated with MIIP-downregulated tumor supernatant exhibited aggravated browning and lipolysis. This finding was further confirmed in subcutaneously allografted mice co-injected with adipocytes and MIIP-downregulated murine CRC cells. Mechanistically, MIIP interacted with the critical lipid mobilization factor AZGP1 and regulated AZGP1’s glycosylation status by interfering with its association with STT3A. MIIP downregulation promoted N-glycosylation and over-secretion of AZGP1 in tumor cells. Subsequently, AZGP1 induced adipocyte browning and lipolysis through the cAMP-PKA pathway, releasing free fatty acids (FFAs) into the microenvironment. These FFAs served as the primary energy source, promoting CRC cell proliferation, invasion, and apoptosis resistance, accompanied by metabolic reprogramming. In a tumor-bearing mouse model, inhibition of β-adrenergic receptor or FFA uptake, combined with oxaliplatin, significantly improved therapeutic efficacy in CRC with abnormal MIIP expression.

**Conclusions:**

Our data demonstrate that MIIP plays a regulatory role in the communication between CRC and neighboring adipose tissue by regulating AZGP1 N-glycosylation and secretion. MIIP reduction leads to AZGP1 oversecretion, resulting in adipose browning-induced CRC rapid progression and poor prognosis. Inhibition of β-adrenergic receptor or FFA uptake, combined with oxaliplatin, may represent a promising therapeutic strategy for CRC with aberrant MIIP expression.

**Supplementary Information:**

The online version contains supplementary material available at 10.1186/s13578-023-01179-0.

## Background

Colorectal cancer (CRC) ranks as the third most prevalent cancer globally. Despite some advancements in screening and therapy, CRC still poses high incidence and mortality rates [1]. Notably, abundant peri-cancerous adipose tissue is a distinct feature of CRC, escalating disease risk and worsening outcomes [2]. During tumor progression, CRC cells infiltrate surrounding adipose tissues and engage in vital communication with adipocytes, which significantly affects prognosis [3]. Cancers can induce metabolic reprogramming in neighboring noncancerous cells, providing the necessary energetic substrates and metabolites for rapid tumor growth [4]. Adipose tissue is a key component of the colorectal tumor microenvironment (TME), and growing evidence suggests that peri-cancerous adipose tissue profoundly influences CRC behavior. Adipocytes have been reported to facilitate colorectal cancer cell proliferation [5], metastasis [6], chemoresistance [7], and stemness [8] through the secretion of cytokines [6] or exosomes [7] and upregulation of metabolic enzymes [8]. However, the intricate and reciprocal interplay between CRC and adipose tissue still requires further elucidation.

Numerous studies indicate that several cancers induce metabolic reprogramming of white adipose tissue (WAT) via browning [9]. The β-adrenergic receptor (β-AR) and AMP kinase (AMPK) signaling pathway are crucial for WAT browning [10, 11] and promote the expression of uncoupling protein 1 (UCP1), which shifts mitochondrial electron transport from ATP synthesis to thermogenesis, leading to increased lipid mobilization and energy expenditure [12]. Consequently, brown adipocytes possess numerous mitochondria and numerous small cytoplasmic droplets compared to white adipocytes [9]. Lipolysis, closely linked to WAT browning, is regulated by three essential lipases, patatin-like phospholipase domain-containing 2 (PNPLA2, also known as ATGL), hormone-sensitive lipase (HSL), and monoglyceride lipase (MGLL) [13]. Patients with cancer have exhibited increased HSL mRNA and protein levels in WAT, leading to enhanced lipolytic activity and elevated serum levels of free fatty acids (FFAs) while reducing body fat [14, 15]. This augmented lipolysis is associated with abnormal secretion of inflammatory peptides, leading to stromal cell, macrophage, and lymphocyte infiltration, significantly altering the microenvironment [16]. Tumor-derived factors such as IL-6, TNF-α, IFN-γ, AZGP1, and PTHrP, and tumor-host interactions influence metabolic programs in adipose tissue, including browning, lipolysis, inflammation, and thermogenesis [17]. Nonetheless, the impact of tumor cells on adjacent adipose tissues in CRC and the underlying regulatory mechanisms require further elucidation.

Migration and invasion inhibitory protein [MIIP, also known as IIp45] was initially identified as a binding partner of insulin-like growth factor-binding protein 2 (IGFBP2) and a negative regulator of cell invasion in glioma [18]. Increasing evidence suggests that MIIP is downregulated in various cancer types [19–21]. Previous studies focusing on urinary system tumors revealed that MIIP directly interacts with PP1α, negatively regulating the Akt pathway, and inhibiting prostate cancer proliferation [22]. The MIIP-miR-181a/b-5p-KLF17 axis has been shown to inhibit prostate cancer epithelial-mesenchymal transition (EMT) [23]. Additionally, MIIP promotes HIF-2α ubiquitination, inhibiting clear-cell renal cell carcinoma proliferation and angiogenesis [24]. These findings indicate that MIIP plays an extensive and effective tumor-suppressive role. However, its role in the interplay between CRC and peri-cancerous adipose tissue and the regulation of WAT browning remain to be determined.

In this study, we compared MIIP expression and surrounding WAT in human CRC tissue samples with different differentiation statuses, finding that MIIP expression was significantly decreased in high-grade CRC samples and inversely correlated with WAT browning and lipolysis. To analyze communication between MIIP-downregulated tumors and adipocytes, we established in vitro and in vivo models and demonstrated that MIIP down-regulated tumor cells enhanced adipocyte browning and lipolysis, as well as the tumor-promoting function of adipocytes by releasing FFAs into the TME. Through RNA sequencing, LC–MS/MS, and co-immunoprecipitation, we further showed that MIIP directly bound to the lipid mobilization factor AZGP1, regulating its secretion. MIIP downregulation and subsequent AZGP1 secretion disorder contributed to abnormal communication between CRC and surrounding adipose tissues. These data provide new insights into the tumor-suppressive role of MIIP and explain the mechanism by which abnormal communication between tumor cells and adipocytes induced by MIIP downregulation promotes CRC progression.

## Results

### MIIP expression was downregulated in CRC tissues

**Fig. 1:**
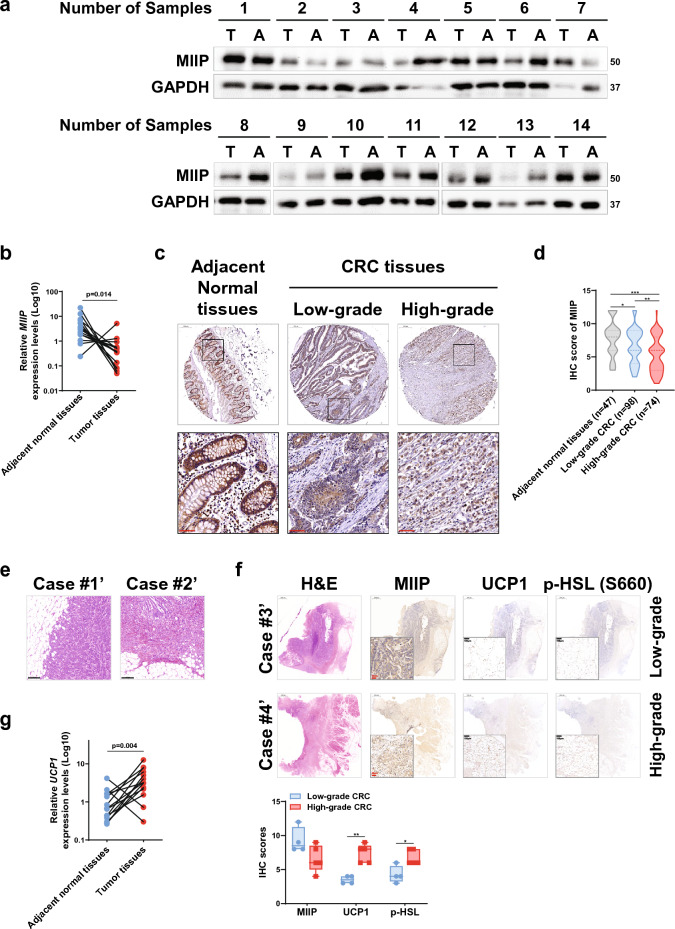
MIIP is found to be downregulated in colorectal cancer (CRC) samples, showing an inverse correlation with the browning of peri-cancerous adipose tissue. **a** Immunoblots were performed to assess MIIP protein levels in 14 paired colorectal cancer samples and adjacent normal tissues from human patients. T represents tumor tissue, and A represents adjacent normal tissue. **b** RT-qPCR was used to detect the MIIP mRNA level in clinical colorectal cancer samples described in **a**; the P-value was calculated using a stratified log rank test (n = 14). **c** Immunohistochemical staining on tissue microarrays (TMAs) composed of colorectal cancer and adjacent normal tissues was used to evaluate MIIP expression. Representative images are shown, with a scale bar of 200 μm (black/top) and 50 μm (red/bottom). **d** Statistical analysis of MIIP expression in CRC tissues and adjacent normal tissues was performed using IHC staining on the TMA shown in **c**. The staining score was calculated by multiplying the stained area (%) score (≤ 5%: 0, 6–25%: 1, 25–50%: 2, 51–75%: 3, ≥ 75%: 4) and the intensity score (colorless: 0, mild: 1, moderate: 2, strong: 3). High expression was specified as a score greater than or equal to 9, while low expression corresponded to a score less than 9. All scoring work was performed independently by two pathologists. **e** Representative images of H&E staining on clinical CRC samples are shown (Scale bar: 200 μm). The tumor growth exposes it to the adjacent adipose tissue, mainly composed of adipocytes. **f** IHC staining was used to assess the endogenous levels of MIIP, UCP1, and p-HSL in clinically defined human colorectal cancer samples. Statistical analysis was also conducted. **g** UCP1 mRNA level was detected in clinical colorectal cancer samples and paired adjacent normal tissues described in **a**–**b** (n = 14). Grade refers to WHO histological grade. *P < 0.05, **P < 0.01, ***P < 0.001

MIIP has been identified as downregulated in various types of tumors, and its functional roles and clinical significance have been demonstrated in prostate cancer and clear cell renal cell carcinoma in our previous studies [22–24]. Similar findings were observed in CRC, consistent with previous results [19]. However, the precise mechanism of its action requires further elucidation. To address this, we investigated MIIP protein levels in 14 paired samples of CRC, comparing tumor and adjacent normal tissues. We detected downregulation of MIIP protein levels in more than half (8/14, Samples #4, #6, #8–13) of the tumor samples compared to the corresponding normal tissues (Fig. 1a). Furthermore, MIIP mRNA levels showed a significant reduction in most of the tumor samples compared to adjacent normal samples (Fig. 1b), indicating decreased MIIP expression in CRC tissues.

Subsequently, we analyzed MIIP expression in tissue microarrays (TMAs) consisting of 172 samples from CRC patients (148 adenocarcinomas and 24 mucinous adenocarcinomas) and 47 samples from matched adjacent normal tissues using IHC staining. We observed that MIIP protein levels were diminished in CRC tissues, especially in high-grade tumors, where MIIP levels were significantly downregulated (Fig. 1c, d). Based on the staining scores of MIIP in tissues, we divided the specimens into a low-MIIP group and a high-MIIP group. Interestingly, 42.6% (20/47) of adjacent normal tissues exhibited high MIIP expression, while only 28.6% (28/98) of low-grade CRC tissues (including well- and moderate-differentiated samples) and 18.9% (14/74) of high-grade CRC tissues (i.e. poorly-differentiated samples) showed high MIIP expression (Additional file 1: Fig. S1a). These findings further confirm the downregulation of MIIP expression in CRC compared to adjacent normal tissues, with a further reduction in high-grade CRC tissues.

### WAT adjacent to high-grade CRC showed evident browning

To explore the relationship between MIIP downregulation and the clinicopathological grade of CRC, we conducted hematoxylin and eosin staining analysis on a series of paraffin-embedded clinical CRC samples. The analysis revealed a significant enrichment of WAT in many patients (Fig. 1e), consistent with previous findings [8, 25]. Notably, different tumor types have been known to promote the browning of adjacent WAT [26–28]. Therefore, we investigated the browning of WAT in clinical CRC samples. Using the browning marker UCP1, we observed that adipose browning was exacerbated in high-grade tumors but exhibited an inverse correlation with MIIP expression (Fig. 1f). Additionally, the activation of HSL, a key enzyme involved in lipolysis, was enhanced in high-grade tumors (Fig. 1f), hinting at a potential link between MIIP abundance, adipocyte browning, and CRC progression.

Subsequently, we assessed UCP1 mRNA levels in tumor tissues and adjacent normal tissues in paired clinical samples using RT-qPCR. The results demonstrated a significant increase in UCP1 expression levels in CRC tissues compared to adjacent normal tissues (Fig. 1g), which contrasted with the downregulated MIIP levels (Fig. 1b). These findings further support the notion that WAT adjacent to high-grade CRCs undergoes noticeable browning, and the downregulation of MIIP may play a role in this process.

### MIIP downregulation in CRC cells exacerbated adipocyte browning

**Fig. 2:**
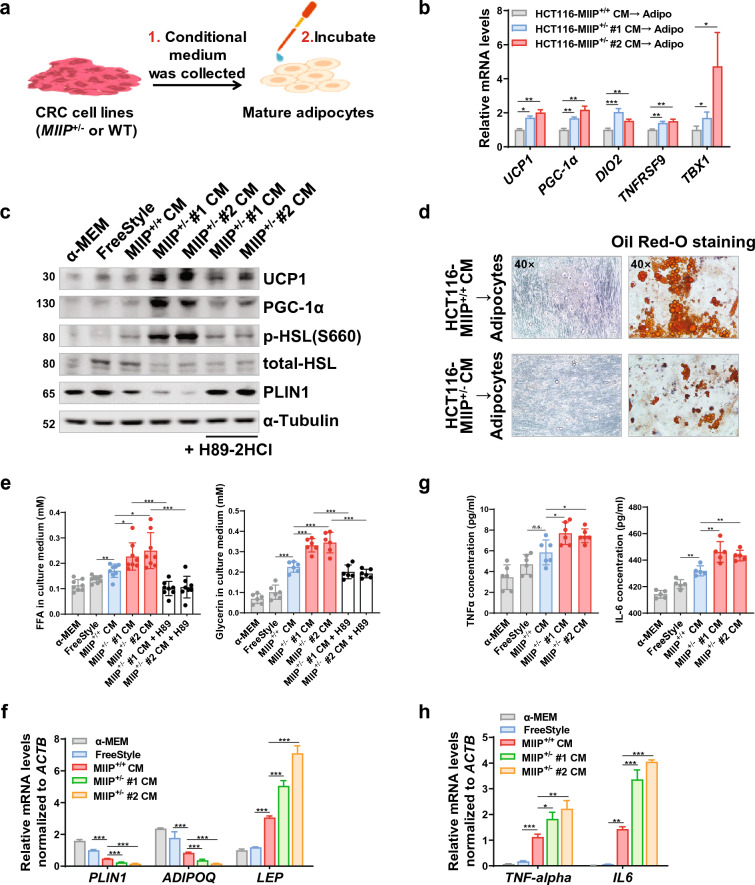
Conditioned medium (CM) derived from MIIP-downregulated CRC cells induces increased browning, lipolysis, and cytokine secretion in adipocytes. **a** The flowchart outlines the preparation of CM from HCT116 WT or MIIP^+/−^ cells and the subsequent treatment of differentiated mature adipocytes. **b** RT-qPCR analysis reveals the expression of browning-related genes in mature adipocytes after 24 h of treatment with CM from HCT116 (MIIP^+/−^ or WT) cells (Adipo: mature adipocytes; 6 biological replicates per group). **c** Immunoblots show the levels of mitochondrial proteins (UCP1, PGC-1α), a key enzyme in lipolysis (total/phospho-HSL), and a lipid droplet protective protein (PLIN1) in mature adipocytes treated with CM from HCT116 (MIIP^+/−^ or WT) cells for 24 h (H89-2HCl: 20 μM for 3 h; representative of 3 biological replicates per group). **d** Representative images of optical microscope and Oil Red O staining in mature adipocytes treated with CM from HCT116 (MIIP^+/−^ or WT) cells for 24 h (40 × for images with a 50 μm scale bar; 3 biological replicates per group). **e** Mature adipocytes were treated with the indicated CM for 24 h, then washed, and the medium was changed. After another 24 h, the concentrations of free fatty acids (Left, 8 biological replicates per group) and glycerol (Right, 6 biological replicates per group) in supernatants were detected (H89-2HCl: 20 μM for 3 h). **f** RT-qPCR analysis shows the expression of white adipocyte-related genes in mature adipocytes after the treatment described in **e** (6 biological replicates per group). **g** Concentrations of TNFα (Left, 6 biological replicates per group) and IL-6 (Right, 5 biological replicates per group) were measured in supernatants of mature adipocytes described in **e**. **h** RT-qPCR analysis shows the expression of TNFα and IL-6 mRNA in mature adipocytes after the treatment as described in **e** (6 biological replicates per group). All data are presented as mean ± SD. *P < 0.05, **P < 0.01, ***P < 0.001

We collected conditioned medium (CM) from HCT116 cells with MIIP haploinsufficiency (MIIP^+/−^, where MIIP expression was stably downregulated, Additional file 1: Supplementary Figures, Fig. S1b) and corresponding wild-type cells (WT, MIIP^+/+^) to treat mature white adipocytes and determine whether MIIP downregulation mediated the browning of peri-cancerous adipocytes (Fig. 2a). We first isolated human primary adipose-derived stem cells (hADSCs) from abdominal or inguinal subcutaneous fat tissue. ADSCs were identified as CD105^+^/CD34^−^/CD45^−^ cells using flow cytometry (percentage of positive cells: 96.65 ± 1.31%, Additional file 1: Fig. S1c). After adipogenic differentiation, we obtained and identified mature white adipocytes using Oil Red O staining (Additional file 1: Fig. S1d).

The expression levels of genes involved in thermogenesis, including UCP1, PGC-1α, and DIO2, and beige-selective genes, such as TNFRSF9 and TBX1, were significantly higher in adipocytes treated with MIIP^+/−^ cell CM than in those treated with CM from WT HCT116 (Fig. 2b). Additionally, the activation of HSL mildly increased upon WT cell CM treatment, but was significantly enhanced in adipocytes treated with MIIP^+/−^ cell CM (Fig. 2c). However, the levels of perilipin1 (PLIN1), a protective protein of lipid droplets, dramatically decreased at both the protein and mRNA levels in MIIP^+/−^ cell CM–treated adipocytes compared to WT cell CM (Fig. 2c, f). Given that HSL and PLIN1 are mainly regulated by the cAMP–PKA signaling pathway [29], we applied H89-2HCl, a specific PKA inhibitor, to repress the pathway and found that it efficiently suppressed MIIP^+/−^ CM-mediated increases in UCP1 and PGC-1α protein levels and HSL activation, and restored the expression of PLIN1 (Fig. 2c).

Furthermore, we observed a clear reduction in the numbers and volume of lipid droplets in MIIP^+/−^ cell CM–treated adipocytes (Fig. 2d), accompanied by a significant increase in the release of FFAs and glycerol in MIIP^+/−^ CM-incubated adipocytes compared to WT CM-incubated cells (Fig. 2e). Notably, these effects could be reversed by H89-2HCl treatment (Fig. 2e). These findings strongly suggest that MIIP-downregulated cell CM promotes adipocyte browning and lipolysis.

Considering the potential impact of browning on adipocyte secretion profiles, we investigated whether the expression of typical adipokines, such as adiponectin and leptin, and cytokines, namely TNF-α and IL-6, was altered in response to MIIP^+/−^ or WT CM treatment. Our findings revealed a significant decrease in adiponectin levels (encoded by *ADIPOQ*), which are known for their inhibitory effects on cancer [30], while there was a marked increase in leptin levels (encoded by *LEP*), known for their growth-promoting effects [31], in MIIP^+/−^ CM-treated adipocytes (Fig. 2f). Furthermore, the expression of inflammatory cytokines TNF-α and IL-6 was notably upregulated at both protein and mRNA levels upon MIIP^+/−^ CM treatment (Fig. 2g, h). Collectively, these results suggest that MIIP downregulation exacerbates the browning and lipolysis of peri-cancerous adipocytes, subsequently leading to the production of inflammatory cytokines and lipid metabolites.

### MIIP regulated adipocyte browning by inhibiting AZGP1 secretion

**Fig. 3:**
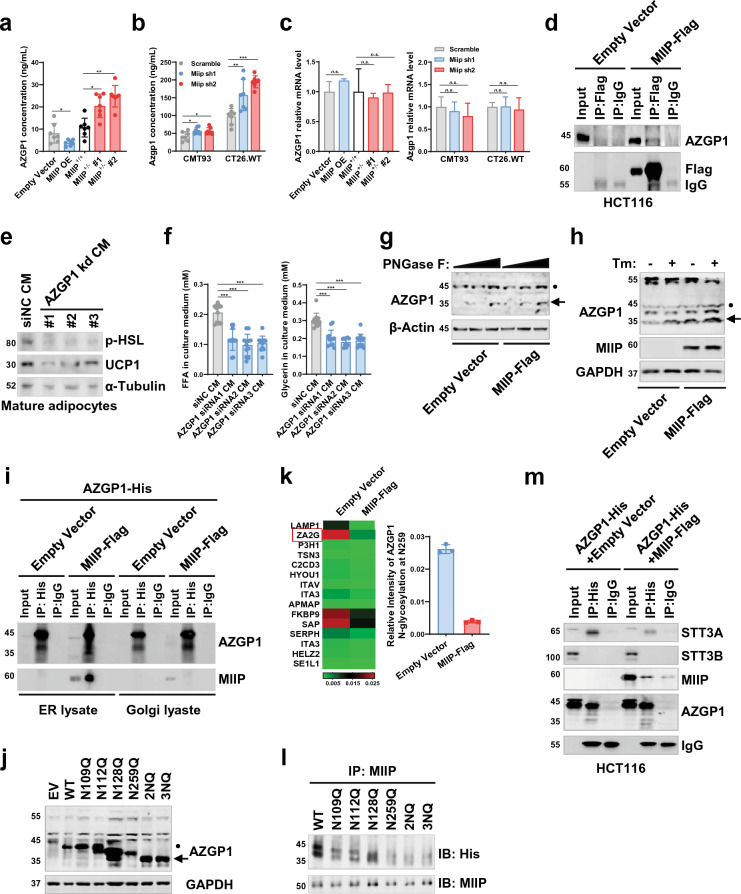
AZGP1 secretion is inversely correlated with MIIP expression. **a**–**b** The concentration of AZGP1 in the supernatant of HCT116 cells **a** and *Miip* stable knockdown CMT93 or CT26.WT cells **b** was detected by ELISA (7 biological replicates per group). **c** The mRNA levels of AZGP1/Azgp1 in the indicated cells were determined by RT-qPCR (3 biological replicates per group). **d** Co-immunoprecipitation analysis of MIIP and AZGP1 in HCT116 cells transfected with pCMV-MIIP-Flag, by immunoprecipitation with anti-Flag and immunoblot with anti-AZGP1. **e** Mature adipocytes were treated with the CM from AZGP1-knockdown or NC HCT116-MIIP^+/−^ cells for 24 h, then washed, and the medium was changed. After an additional 24 h, protein samples were extracted and subjected to immunoblots (Representative of 3 biological replicates per group). **f** The concentration of free fatty acids (Left) and glycerol (Right) in supernatants of cells described in **e** was detected (12 biological replicates per group). **g** Glycosylation pattern of AZGP1 protein in HCT116 control and MIIP-overexpressing cells. Cell lysates were treated with PNGase F and subjected to immunoblot analysis (Representative of 3 biological replicates per group). **h** Glycosylation of AZGP1 protein in HCT116 control and MIIP-overexpressing cells. Cells were treated with Tunicamycin (1 μg/mL) or not and subjected to immunoblot analysis (Representative of 3 biological replicates per group). **i** HCT116 cells were co-transfected with pEX-3-AZGP1-6 × His and pCMV-MIIP-Flag (or empty vector) for 48 h, then ER and Golgi apparatus protein were extracted, followed by co-immunoprecipitation analysis. The lysates were precipitated with anti-His and immunoblotted with anti-AZGP1 and anti-MIIP. **j** Immunoblot analysis of the protein expression pattern of AZGP1 WT and its NQ mutants in HEK-293 cells. **k** LC–MS/MS-based detection of the relative N-glycosylation intensity of AZGP1 at the N259 residue in MIIP-overexpressing cells and control cells, Fold Change = 0.149 (Case/Control, 3 biological replicates per group). **l** HCT116 cells were transfected with the ectopic expression plasmid of AZGP1 WT and its NQ mutants for 48 h, followed by co-immunoprecipitation analysis with anti-MIIP and anti-His successively. **m** Co-immunoprecipitation analysis of MIIP-AZGP1-STT3A/STT3B interaction in HCT116 cells co-transfected with pCMV-MIIP-Flag and pEX-3-AZGP1-6 × His. The lysates were precipitated with anti-His and immunoblotted with anti-AZGP1, anti-MIIP, anti-STT3A, and anti-STT3B. Black spot, glycosylated AZGP1; arrowhead, non-glycosylated AZGP1. All data are presented as mean ± SD. *P < 0.05, **P < 0.01, ***P < 0.001

In previous studies, it was suggested that certain factors secreted by tumors may activate white adipocyte browning through cAMP-PKA signaling [32]. Therefore, we examined the secretory protein profile in MIIP^+/−^ CM and WT CM. Among the differentially secreted proteins, alpha-2-glycoprotein 1, zinc-binding (AZGP1), also known as ZA2G, caught our attention (Additional file 1: Fig. S2a, Additional file 2: Table S1). AZGP1 plays a crucial role in lipid mobilization as it promotes the activation of triacylglycerol (TAG) lipase, leading to increased lipolysis. Notably, AZGP1 has been linked to cancer cachexia [33–35], and TCGA datasets revealed elevated mRNA expression in colon adenocarcinoma (COAD) and rectum adenocarcinoma (READ) (Additional file 1: Fig. S2b). In our experiments, AZGP1 secretion was significantly increased in HCT116-MIIP^+/−^ cells, while it decreased in HCT116 cells with stable MIIP overexpression (Fig. 3a). Similarly, stable Miip knockdown in mouse CRC cell lines, CT26.WT and CMT93, also yielded similar results (Fig. 3b and Additional file 1: Fig. S2c), implying an inverse correlation between MIIP expression level and AZGP1 secretion.

Subsequently, we delved into the mechanism by which MIIP regulates AZGP1 secretion. Surprisingly, we observed that MIIP did not have an impact on the expression level of AZGP1 (Fig. 3c). To gain further insights into the regulatory roles of MIIP in AZGP1 secretion, we conducted tandem affinity purification and mass spectrometry analysis to identify proteins that associate with MIIP. The results revealed a potential direct interaction between MIIP and AZGP1 (Additional file 1: Fig. S2d, Additional file 2: Table S2). To validate this interaction, co-immunoprecipitation (Co-IP) assays were performed in HCT116 cells, demonstrating the specific binding of MIIP to AZGP1 (Fig. 3d). This interaction between MIIP and AZGP1 was further confirmed in HepG2 and T47-D cells, both of which exhibit high endogenous expression of AZGP1 (Additional file 1: Fig. S2e, f).

To investigate the role of AZGP1 in adipocyte lipolysis and browning, we utilized siRNA-induced knockdown of AZGP1 in MIIP^+/−^-HCT116 cells (Additional file 1: Fig. S2g). Subsequently, we assessed the effects on adipocytes after incubating them with CM from AZGP1-silenced CRC cells. The results showed a notable reduction in hormone-sensitive lipase (HSL) activation and uncoupling protein 1 (UCP1) expression in adipocytes exposed to AZGP1 knockdown CM compared to those treated with siNC CM (Fig. 3e). Consequently, there was a decrease in the release of free fatty acids (FFAs) and glycerol from these adipocytes (Fig. 3f). These findings indicate that AZGP1, secreted by colorectal cancer cells, plays a significant role in adipocyte lipolysis and browning.

Given the essential role of N-linked glycosylation in AZGP1 secretion [34, 36], we aimed to investigate whether MIIP downregulation regulates AZGP1 secretion by affecting its N-glycosylation. To explore this, MIIP-overexpressing HCT116 cells were treated with recombinant glycosidase (peptide-N-glycosidase F; PNGase F) to remove the entire N-glycan structure. The cell lysates were then subjected to immunoblot analysis, revealing a significant reduction in the molecular weight of AZGP1 from 45 to 35 kDa in MIIP-overexpressing cells upon administering a lower dose of PNGase F (Fig. 3g). However, no such effect was observed when the cells were treated with O-glycosidase (Additional file 1: Fig. S3a). Moreover, in MIIP-overexpressing cells, endogenous AZGP1 glycosylation was inhibited, resulting in a decrease in molecular weight and increased intracellular retention, even without the treatment of the N-glycosylation inhibitor tunicamycin (Tm), in comparison to the control HCT116 cells (Fig. 3h). Consequently, the secretion of AZGP1 was restrained (Additional file 1: Fig. S3b). These results collectively demonstrate that AZGP1 with the higher molecular weight indeed represents the N-glycosylated form.

As it is widely understood, secretory proteins undergo transportation to the extracellular compartment after processing through the endoplasmic reticulum (ER) and Golgi apparatus. To investigate whether MIIP and AZGP1 interact during this crucial process, we examined HCT116 cells with ectopic expression of MIIP and AZGP1. The ER and Golgi proteins were isolated and identified using the ER marker GRP94 and the Golgi apparatus marker TGN46, respectively (Additional file 1: Fig. S3c). Subsequently, Co-IP assays revealed that the interaction between MIIP and AZGP1 predominantly took place in the ER rather than in the Golgi apparatus (Fig. 3i). Furthermore, we employed immunofluorescence to verify the localization of MIIP and AZGP1 in the ER of both HCT116 and HepG2 cells (Additional file 1: Fig. S3d).

N-glycosylation typically occurs at asparagine residues within the Asn-X-Ser/Thr (NXS/T) consensus motif. Utilizing the online database NetNGlyc-1.0 (https://services.healthtech.dtu.dk/service.php?NetNGlyc-1.0), which identifies N-linked glycosylation sites in human proteins, we identified four potential modification sites within the AZGP1 amino acid sequence (Additional file 1: Fig. S3e, f). To pinpoint the critical site(s) responsible for N-glycosylation within AZGP1, we generated several His-tagged AZGP1 mutants, wherein each of the four asparagines (N) was substituted with glutamine (Q). Subsequently, we observed that N128Q or N259Q led to a slight reduction in glycosylation, resulting in a decrease in molecular weight compared to wild-type (WT) AZGP1 (Fig. 3j). No discernible differences in molecular weight were found for the N109Q and N112Q mutants (Fig. 3j). Interestingly, AZGP1 glycosylation was completely abolished in the 2NQ (N128Q/N259Q) and 3NQ (N112Q/N128Q/N259Q) mutants (Fig. 3j), suggesting that N128 and N259 residues are likely the main sites of N-glycosylation within AZGP1.

To identify the glycan structure, we conducted liquid chromatography coupled with tandem mass spectrometry (LC–MS/MS) analysis on purified human AZGP1 from MIIP-overexpressing and control cells. We detected glycopeptides carrying N-glycans, including the glycan composition and relative intensity of modification. Comparing the results from control cells, the relative modification intensity of N-glycosylation at N259 residue of AZGP1 was notably reduced in MIIP-overexpressing cells (Fig. 3k, Additional file 2: Table S3). However, there was no significant difference in the glycan composition, which consisted of N-acetylglucosamine (GlcNAc) and mannose in both cell types (Additional file 1: Fig. S3g). To further validate whether the N259 residue of AZGP1 is crucial for MIIP binding, we performed Co-IP assays in HCT116 cells that ectopically expressed the aforementioned His-tagged AZGP1 mutants. The results revealed a distinct decline in the interaction between MIIP and N259 mutant AZGP1 (N259Q/2NQ/3NQ), indicating that N259 residue is critical for AZGP1 and MIIP interaction (Fig. 3l). In conclusion, our findings demonstrate that AZGP1 undergoes exclusive N-glycosylation at N128 and N259 residues. MIIP directly binds to N259 of AZGP1 in the ER, thereby impeding its secretion by inhibiting N-glycosylation.

### MIIP bound to AZGP1 and inhibited its N-glycosylation through competing its association with STT3A

In view of the significance of N-glycan biosynthesis in influencing N-glycosylation, our investigation aimed to determine whether MIIP (a specific protein) played a role in this process. To explore this, we conducted RNA sequencing (RNA-seq) analysis on HCT116-MIIP^+/−^ cells in comparison with WT cells. The gene set enrichment analysis revealed no significant difference in N-glycan biosynthesis-related genes between MIIP^+/−^ and WT cells (Additional file 1: Fig. S4a). The N-oligosaccharyl-transferase (OST) complex plays a pivotal role in the proper folding of nascent polypeptide chains within the endoplasmic reticulum (ER). Within this complex, STT3A and STT3B serve as two distinct catalytic subunits responsible for transferring high-mannose oligosaccharides from lipid-linked oligosaccharide donors to asparagine residues within the NXS/T consensus motif of nascent polypeptide chains [37, 38]. In light of this, we conducted RNA-seq and RT-qPCR analyses to assess the potential impact of MIIP overexpression or knockdown on the expression of STT3A and STT3B in both human and mouse colorectal cancer (CRC) cell lines. However, our findings from these analyses demonstrated no significant effects of MIIP on the expression of STT3A and STT3B (Additional file 1: Fig. S4b–e). As a result, we concluded that MIIP does not appear to be involved in N-glycan synthesis and does not alter the expression of glycosyltransferases.

Consequently, we postulated that MIIP could potentially regulate N-glycosylation by influencing the interaction between STT3A or STT3B and AZGP1. To explore this hypothesis, we conducted Co-IP assays in HCT116 and HepG2 cells overexpressing AZGP1. Remarkably, the ectopic expression of MIIP led to a significant reduction in the binding of STT3A to AZGP1 (Fig. 3m and Additional file 1: Fig. S4f). This observation indicated that MIIP might competitively bind to the AZPG1-STT3A complex, thus inhibiting STT3A-mediated glycosylation of AZGP1. However, no detectable interaction was observed between AZGP1, STT3B, and MIIP (Fig. 3m and Additional file 1: Fig. S4f).

AZGP1 is recognized for its ability to activate the cAMP-PKA signaling pathway by binding to a β3-adrenergic receptor, resulting in lipolysis and browning [34]. To investigate further, we treated mature adipocytes with a specific inhibitor of β-adrenergic receptors, SR59230A, in the presence of CM from MIIP^+/−^ or WT cancer cells. Notably, the decreased numbers and volume of lipid droplets caused by MIIP^+/−^ CM were significantly restored (Additional file 1: Fig. S5a). Moreover, we conducted experiments where adipocytes were incubated with CM from MIIP^+/−^ or WT cancer cells, and β-adrenergic receptor blockade was achieved using either SR59230A or H89-2HCl. The results demonstrated a substantial reduction in the phosphorylation of PKA substrate proteins and a downregulation of UCP1 and PGC-1α levels (Additional file 1: Fig. S5b). However, it is important to note that the inflammatory pathways (JAK-STAT and NF-κB), proliferation (ERK1/2), and apoptosis (PARP and caspase-3) were not significantly affected by the incubation of MIIP^+/−^ CM and the blockade of β-adrenergic receptors (Additional file 1: Fig. S5c).

Collectively, these findings suggest that MIIP hinders the N-glycosylation of AZGP1 by interacting with AZGP1 and competing for its binding with STT3A, consequently leading to a reduction in its secretion. On the other hand, downregulation of MIIP leads to an augmented secretion of AZGP1, which, in turn, triggers WAT lipolysis and browning through the activation of the β-adrenergic receptor-cAMP-PKA pathway.

### MIIP downregulation led to increased browning of peri-cancerous WAT in allografted mice

**Fig. 4:**
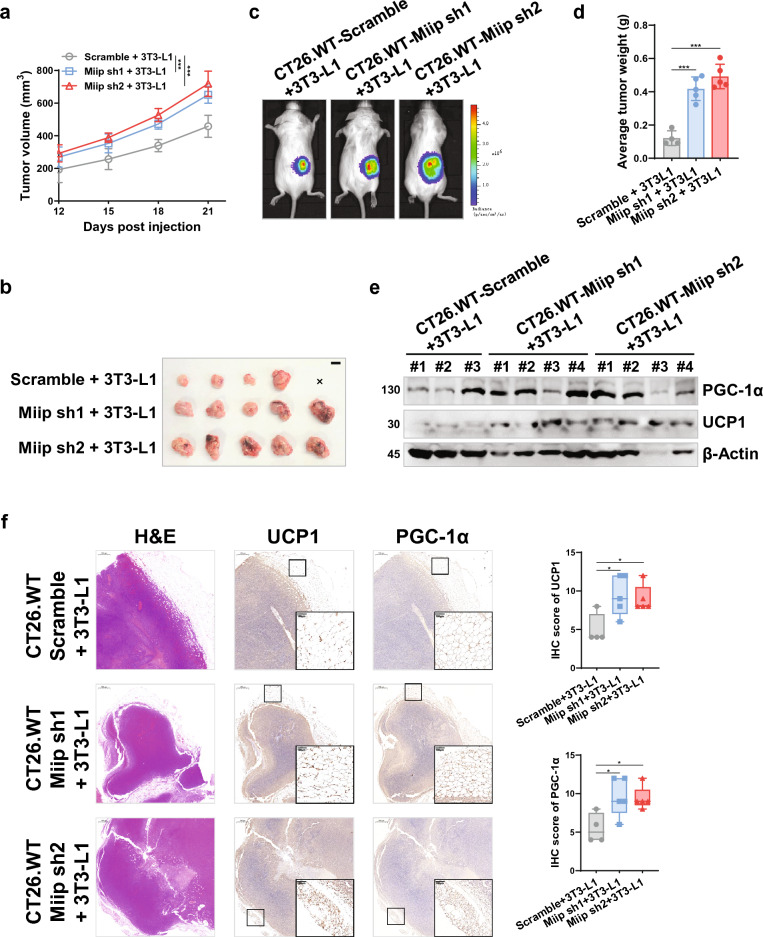
Miip downregulated cancer cells aggravate the browning of tumor-adjacent adipocytes in vivo. **a**–**b** Stable *Miip*-knockdown or scramble CT26.WT cells carring LUC (2 × 10^5^ cells per mouse) were co-injected unilaterally with 3T3-L1 cells (5 × 10^4^ cells per mouse) into the back of BALB/c mice. Tumor growth was monitored every 3 days **a**, and the tumors were removed (**b**, scale bar: 1 cm) at the end of the experiment (n = 5). **c** Representative bioluminescence images of tumor growth in mice described in **a**, **b** at day 21 (n = 5). **d** The tumors described in **b** were weighed at the end of the experiment. **e** Immunoblots of UCP1 and PGC-1α proteins in allograft tumors derived from **b**. **f** Representative images and statistical analysis of UCP1 and PGC-1α proteins in allograft tumors derived from **b** by IHC staining (n = 5, scale bar: 100 μm). All data are presented as mean ± SD. *P < 0.05, ***P < 0.001

To validate the impact of MIIP downregulation in CRC cells on the browning of peri-cancerous adipocytes in vivo, CRC cell allografts were generated. For this purpose, murine CRC cell lines (CT26.WT or CMT93) with stable *Miip* knockdown were utilized, as confirmed in Additional file 1: Fig. S2c. These cells, along with 3T3-L1 cells, were subcutaneously injected into the backs of BALB/c or C57B/L6 mice in a unilateral manner, with a 4:1 ratio (Additional file 1: Fig. S6a). Throughout the experiment, we closely monitored the tumor growth and observed that tumors containing *Miip* knockdown CT26.WT cells (Fig. 4a) or CMT93 cells (Additional file 1: Fig. S6b) exhibited a higher growth rate compared to tumors originating from scramble cells. Consequently, tumors derived from a mixture of CRC cells (CT26.WT or CMT93) with Miip knockdown and adipocytes (3T3-L1 cells) were notably larger (Fig. 4b, c and Additional file 1: Fig. S6c, d) and heavier (Fig. 4d and Additional file 1: Fig. S6e) than tumors from the scramble group, particularly during the later stages post-injection. Furthermore, we observed evidence of adipocyte browning in the co-injected 3T3-L1 cells. UCP1 protein expression was notably enhanced in the tumors originating from the mixture of *Miip* knockdown CT26.WT cells (Fig. 4e, f) or CMT93 cells (Additional file 1: Fig. S6f) along with 3T3-L1 cells.

### FFAs released by beige adipocytes promoted CRC cell proliferation, invasion, and survival

**Fig. 5:**
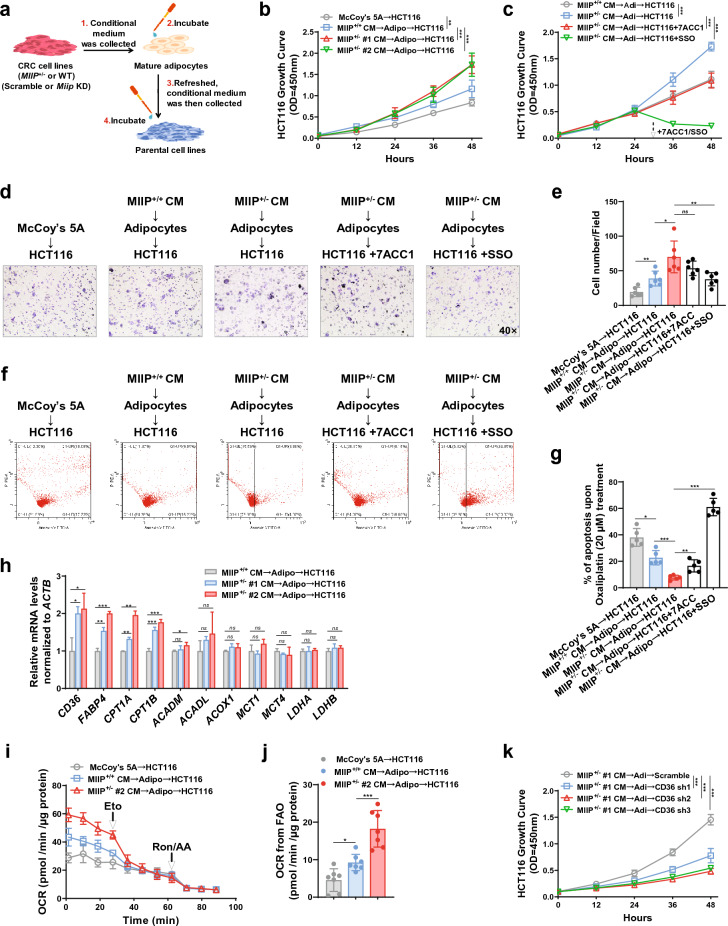
Beige adipocytes released FFAs are essential for CRC proliferation, invasion, and survival. **a** A flowchart illustrating the two-step CM preparation and treatment of human or mouse parental CRC cell lines. 1: CM preparation from MIIP-decreased or control cells; 2: Treatment of mature adipocytes with the corresponding CM; 3: After exposure to CRC CM for 24 h to induce their browning, the adipocytes were washed, and the medium was refreshed; 4: After another 24 h, the adipocytes CM were collected and applied to fresh cultures of parental CRC cell lines. **b** Parental HCT116 cells treated with the two-step CM as described in **a**, and cell viability was measured with CCK-8 (7 biological replicates per group). **c** Parental HCT116 cells treated with the two-step CM as described in **a**, and cell viability was measured with CCK-8, 7ACC1 (10 μM), or SSO (20 μM) were added at the indicated time point, respectively (7 biological replicates per group). **d**–**e** Parental HCT116 cells treated with the two-step CM as described in **a**, and the invasive ability was evaluated by trans-well invasion assay (40 × for images with a 50 μm scale bar. 7ACC1: 10 μM; SSO: 20 μM. 6 biological replicates per group). **f**–**g** Parental HCT116 cells treated with the two-step CM as described in **a**, and cell apoptosis induced by oxaliplatin was determined by PI-Annexin V staining **f** followed by flow cytometric analysis and quantification **g**, 7ACC1: 5 μM, SSO: 10 μM (5 biological replicates per group). **h** RT-qPCR-determined expression of genes related to FFAs transport (CD36 and FABP4), FAO, lactate transport (MCT1 and MCT4), and catabolism (LDHA and LDHB) in parental HCT116 cells treated with the two-step CM as described in **a** (6 biological replicates per group). **i** Oxygen consumption rates (OCR) were measured by Seahorse XF analysis in parental HCT116 cells at 24 h after exposure to the indicated CM. Arrows indicate the time when Etomoxir (Eto, 50 μM), and rotenone (Ron, 0.2 μM)/antimycin A (AA, 0.2 μM) were added (7 biological replicates per group). **j** The amount of OCR derived from FAO of HCT116 cells treated with the indicated CM (1 technical replicate of 7 biological replicates per group). **k** Stable *CD36*-knockdown or scramble HCT116 cells treated with the two-step CM as described in **a**, and cell viability was measured with CCK-8 (7 biological replicates per group). Adi or Adipo: mature adipocytes. All data are presented as mean ± SD. *P < 0.05, **P < 0.01, ***P < 0.001

Considering their close proximity to tumors, we investigated whether the browning of tumor-adjacent adipocytes might facilitate CRC progression. To explore this, we conducted a series of parallel two-stage experiments involving the treatment of conditioned media (CM) derived from MIIP-downregulated CRC cells (MIIP^+/−^ and Miip shRNA) and control cells (MIIP^+/+^ and scramble). These experiments were designed to simulate the putative bi-directional communication between CRC cells and adipocytes (Fig. 5a). In detail, we first pretreated mature adipocytes with CM from MIIP-downregulated or control CRC cells to induce their browning. Subsequently, we applied the adipocyte CM to fresh cultures of human or mouse parental CRC cell lines (Fig. 5a). The incorporation of CCK8 revealed that CM obtained from adipocytes pretreated with MIIP-downregulated CRC cell CM significantly promoted the proliferation of recipient CRC cells. Conversely, CM obtained from adipocytes pretreated with control CM had only a mild effect on cell proliferation (Fig. 5b and Additional file 1: Fig. S7a). These findings strongly suggest that within the CRC tumor microenvironment, adjacent beige adipocytes possess the capacity to enhance CRC cell proliferation through feedback communication.

To further elucidate the role of peri-cancerous adipocytes in CRC progression, we explored the browning process of adipocytes, which involves the secretion of numerous cytokines and small molecules, including metabolites. Such secreted factors have the potential to beneficially alter the tumor microenvironment (TME). Previous studies have reported the influence of adipose-derived metabolites, particularly FFAs and lactate, in altering the TME and facilitating cancer progression [28, 39, 40]. In light of this, we introduced two inhibitors, the fatty acid transporter inhibitor sulfosuccinimidyl oleate (SSO) and the lactate transporter MCT1/4 inhibitor 7ACC1, to recipient CRC cells in the two-stage CM treatment system. Notably, inhibiting cellular fatty acid transporters with SSO led to a rapid reduction in the proliferation rate of both human and mouse CRC cells. In contrast, 7ACC1, which specifically targeted lactate intake, caused only a slight suppression of proliferation (Fig. 5c and Additional file 1: Fig. S7b, c). Furthermore, through the trans-well assay, we observed a distinct enhancement in recipient cell invasion upon the addition of CM from adipocytes pretreated with MIIP^+/−^ cell CM. However, CM from adipocytes pretreated with WT CM had a modest effect on invasion. Moreover, this effect could be significantly reversed in the presence of SSO, but not 7ACC1 (Fig. 5d, e).

To determine the significance of beige adipocytes in the survival of CRC cells, we assessed oxaliplatin-induced apoptosis in recipient CRC cells treated with two-stage CM. The results, depicted in Fig. 5f, g, and Additional file 1: Fig. S7d, e, revealed a substantial decline in apoptosis in cells incubated with CM from adipocytes pretreated with MIIP-downregulated cell supernatant. In contrast, CM from adipocytes pretreated with control supernatant had a relatively modest effect on cell survival. Interestingly, both 7ACC1 and SSO promoted oxaliplatin-induced apoptosis, with SSO exhibiting a more pronounced effect.

The downregulation of MIIP resulted in a significant increase in the secretion of FFAs from co-cultured adipocytes, as demonstrated earlier (Fig. 3e). This observation suggests that CRC cells may have a greater propensity to uptake and utilize FFAs as their primary fuel source to expedite their evolution, rather than relying on lactate. Additionally, upon performing Oil Red O staining, we observed a higher number of lipid droplets within the cytoplasm of HCT116 cells incubated with CM from adipocytes pretreated with MIIP^+/−^ cancer cell CM, in comparison to cells incubated with CM from WT cells (MIIP^+/+^) or the conventional medium (McCoy’s 5A) (Additional file 1: Fig. S7f, g). These results indicate that the excessive release of FFAs from adjacent white adipose tissue (WAT) may contribute to the increased lipid content in CRC cells, which is consistent with findings from previous reports [41, 42].

Consistent with the increased secretion of FFAs from beige adipocytes in the tumor microenvironment (TME), cells incubated with adipocyte CM pretreated with MIIP^+/−^ cancer cell CM exhibited higher expression levels of FFA transporters, CD36 and FABP4, compared to cells treated with WT (MIIP^+/+^) CM (Fig. 5h). Notably, the expression of genes involved in fatty acid oxidation (FAO), such as CPT1A and CPT1B, both located in the outer mitochondrial membrane and playing a central role in long-chain fatty acyl-CoA transport from the cytoplasm into the mitochondria, was also higher in these cells. However, the expression of other key enzymes, such as ACADM, ACADL, and ACOX1, did not change significantly (Fig. 5h). In contrast, the expression of lactate transporters MCT1 and MCT4 and the key catabolic enzymes LDHA and LDHB did not undergo remarkable changes in cells treated with different CM (Fig. 5h), further confirming the increased uptake and utilization of FFAs in CRC cells. Additionally, Etomoxir (Eto, a CPT1a inhibitor) significantly impaired the OCR (oxygen consumption rate) of parental HCT116 cells incubated with tumor-adipocyte-co-cultured supernatant, particularly in MIIP^+/−^-derived supernatant cultured cells but not in conventional (McCoy's 5A) cultured cells (Fig. 5i, j). Therefore, we inferred that the catabolism of FFAs might be more vigorous in cells incubated with MIIP-downregulated tumor-adipocyte co-cultured supernatant.

To assess the significance of CD36 in fatty acid transportation, we generated HCT116 cells with stable CD36 knockdown and subsequently incubated them with adipocyte CM pretreated with MIIP^+/−^ cancer cell CM. Remarkably, we observed a substantial suppression in the proliferation rate when CD36 expression was reduced (Fig. 5k). These findings strongly suggest that CD36 is essential for the growth facilitation of CRC cells incubated with tumor-adipocyte co-cultured supernatant.

### Crosstalk between MIIP-downregulated CRC cells and adipocytes promoted cancer growth in xenografted mice

**Fig. 6:**
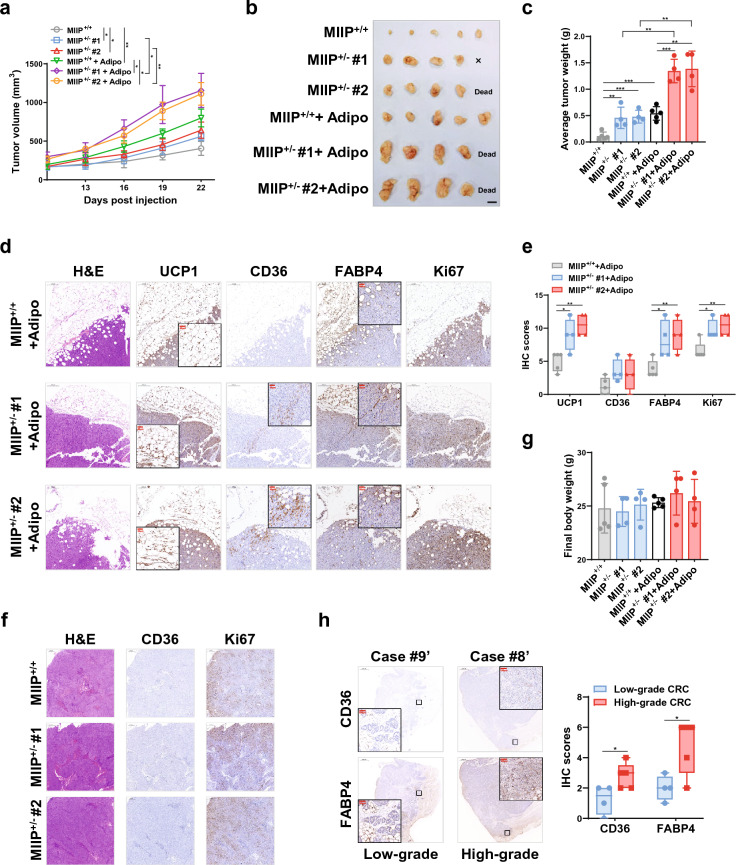
Communication between beige adipocytes and MIIP downregulated CRC cells promotes tumor growth in vivo. **a**–**c** HCT116-MIIP^+/−^ or WT cells (1 × 10^6^ cells per mouse) were co-injected unilaterally with primary adipocytes (2.5 × 10^5^ cells per mouse) into the back of nude mice (n = 5). Tumor growth was monitored every 3 days **a**. The tumors were removed (**b**, scale bar: 1 cm) and weighed (**c**) at the end of the experiment. **d**–**e** Representative images (**d**) and statistical analysis (**e**) of UCP1, CD36, FABP4, and Ki67 proteins by IHC staining and representative images of H&E staining in xenograft tumors derived from (B) (n = 5, scale bar: 50 μm, red). **f** Representative images of IHC staining of CD36 and Ki67 proteins and H and E staining in xenograft tumors derived from mice injected with HCT116 (MIIP^+/−^ or WT) alone (n = 5, scale bar: 200 μm). **g** Body weights of mice monitored in (A) on day 22 (n = 5). **h** Representative images and statistical analysis of endogenous CD36 and FABP4 in clinically defined human colorectal cancer samples by IHC staining (Scale bar: 100 μm, red). Adipo: mature adipocytes. All data are presented as mean ± SD. *P < 0.05, **P < 0.01, ***P < 0.001

To investigate the in vivo effects of communication between MIIP-downregulated CRC cells and adipocytes, we prepared CRC subcutaneous xenografts. HCT116 cells (MIIP^+/−^ or WT) were injected along with mature adipocytes, or HCT116 cells were injected alone, into the back of nude mice at a 4:1 ratio. Consistently, tumors with downregulated MIIP exhibited more rapid growth (Fig. 6a, b). Additionally, tumors originating from mixed CRC and adipocyte cells were larger and heavier than the corresponding control tumors (HCT116-alone, MIIP^+/−^, or WT) 22 days after injection (Fig. 6a–c). As evidence of increased browning of co-injected mature adipocytes, UCP1 protein expression was upregulated in the tumors of mixed HCT116-MIIP^+/−^  + mature adipocyte origin compared to HCT116-MIIP^+/+^  + mature adipocytes (Fig. 6d, e). Consequently, tumors originating from mixed HCT116-MIIP^+/−^ and adipocytes showed higher expression levels of the FFA transporters CD36 and FABP4. This effect was particularly prominent in the area where the tumor was in direct contact with adipocytes, as evidenced by a higher proportion of Ki67-positive cells in that region (Fig. 6d, e). However, we did not detect any significant changes in CD36 abundance in mice injected with HCT116 (MIIP^+/−^ or WT) alone (Fig. 6f), suggesting that CD36 upregulation in tumor cells was attributed to adjacent adipose browning. Moreover, no remarkable differences in final body weight were observed between HCT116 (MIIP^+/−^ or WT) + adipocytes and HCT116-alone (MIIP^+/−^ or WT) groups, indicating that cachexia did not occur during the assays (Fig. 6g).

Subsequently, we conducted IHC staining on CRC samples to assess the expression of CD36 and FABP4 in clinical specimens. Our findings revealed a significant elevation in the levels of both FFA transporters in high-grade CRC tissues (Fig. 6h), which aligns with previous reports indicating that CD36 and FABP4 play a role in promoting colon cancer metastasis and are associated with a poor prognosis [43, 44].

### Inhibition of FFA importation impaired beige adipocyte-induced CRC growth

**Fig. 7:**
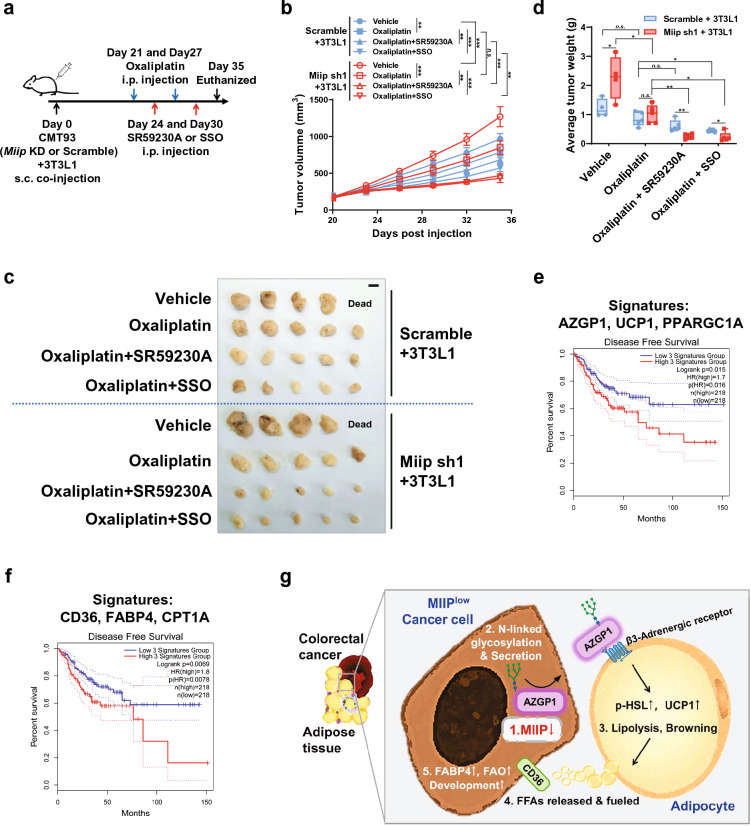
Targeting browning or FFAs uptake enhances the anti-tumor efficacy of oxaliplatin in vivo. **a** Flowchart of constructing the subcutaneous tumor-bearing model and implementing combined therapy in C57B/L6 mice, using oxaliplatin (5 mg/kg), SR59230A (8 mg/kg), and SSO (20 mg/kg) through intraperitoneal injection (i.p.). **b**–**d** Subcutaneous tumor growth curve **b** of CMT93 scramble or stable Miip knockdown cells (1 × 10^5^ cells per mouse) mixed with 3T3-L1 cells (2.5 × 10^4^ cells per mouse). Treatment (vehicle, oxaliplatin, oxaliplatin plus SR59230A or SSO) began when the volume of allografts reached approximately 150 mm^3^. The allograft tumors were removed (**c**, scale bar: 1 cm) and weighed **d** at the end of the experiment (n = 5). **e**–**f** Kaplan–Meier analysis of disease-free survival for gene signatures related to adipocyte browning (AZGP1/UCP1/PPARGC1A, **e** and FFAs transportation and oxidation (CD36/FABP4/CPT1A, **f**) in COAD and READ patients. **g** The graphical abstract describes how abnormal MIIP expression leads to excessive secretion of AZGP1, promoting bi-directional communication between CRCs and surrounding adipose tissue, and the consequent tumor-supportive role of FFAs released from adjacent adipocytes in the TME. All data are presented as mean ± SD. *P < 0.05, **P < 0.01, ***P < 0.001

Subsequently, we aimed to assess the efficacy of conventional chemotherapeutic agents in the treatment of MIIP-downregulated CRC when combined with the inhibition of adipocyte browning or FFA uptake in an in vivo setting. To achieve this, we constructed a tumor-bearing mouse model by subcutaneously co-injecting CMT93 cells (with stable Miip knockdown or scramble) along with 3T3-L1 cells. The mice were then treated with oxaliplatin alone or in combination with either SR59230A or SSO (Fig. 7a). As depicted in Fig. 7b, oxaliplatin alone exhibited partial inhibition of tumor growth, irrespective of the MIIP expression level, compared to the vehicle-treated group. However, the combined therapy (oxaliplatin + SR59230A or SSO) significantly suppressed tumor growth in MIIP-downregulated tumors, resulting in markedly reduced average tumor size and weight compared to oxaliplatin alone treatment and scramble tumors (Fig. 7b–d). The combined therapy demonstrated a partial inhibitory effect in tumors with normal MIIP expression, likely attributed to the tumor-suppressive functions of SR59230A and SSO [45, 46]. However, this effect was limited (Fig. 7b–d), indicating that blocking β-adrenergic receptor–mediated adipocyte browning or FFA uptake by tumor cells could effectively enhance the therapeutic efficiency of oxaliplatin in the treatment of MIIP-aberrant CRC.

Furthermore, we sought to investigate the prognostic value of gene signature groups associated with adipose browning and FFA transportation signaling pathways, which include UCP1, CD36, and FABP4, in a cohort of COAD and READ, based on the GEPIA2 database. The results, as depicted in Fig. 7E, F, revealed that high expression levels of the gene signature group associated with adipose browning (AZGP1, UCP1, and PPARGC1A, Fig. 7e) and FFA transportation and oxidation (CD36, FABP4, and CPT1A, Fig. 7f) were strongly correlated with poor disease-free survival.

## Discussion

Peri-tumoral adipocyte infiltration during the development of CRC is closely associated with rapid progression and poor prognosis. However, the detailed regulatory mechanism remains elusive. In this study, we demonstrated that the bi-directional communication between CRC and adjacent adipose tissue boosts CRC progression, and that MIIP plays a key regulatory role in this process. MIIP down-regulation in colorectal cancer cells led to an increase in AZGP1 N-glycosylation and subsequent aberrant secretion of AZGP1, which, in turn, induced peri-cancerous WAT browning and lipolysis via the cAMP–PKA pathway. The resulting FFAs were then released into the TME and served as the main fuel to facilitate the proliferation, invasion, and apoptosis-resistance of cancer cells. Therefore, we propose a novel mechanism by which aberrant expression of MIIP accelerates the rapid progression of CRC (Fig. 7g). This phenomenon, where reduced MIIP expression in high-grade CRCs promotes the browning of adjacent fat to "feed" themselves, might explain, at least in part, why CRCs tend to communicate with adipose tissue and are associated with poorer prognosis. Although the tumor suppressor function of MIIP has been well studied in various types of cancer, our present study demonstrates for the first time that MIIP plays a role in regulating the secretion of lipid mobilization factors, and its aberrant expression is a key initiator of this loop. Moreover, our results also demonstrate that chemotherapy drugs combined with WAT browning or FFAs uptake inhibitors have an impressive synergistic anti-tumor effect, which provides new ideas for the treatment of CRC with abnormal MIIP expression.

WAT browning is associated with increased expression of UCP1, which uncouples mitochondrial respiration towards thermogenesis instead of ATP synthesis, leading to increased lipid mobilization and energy expenditure [12]. This is distinct from cancer-associated adipocytes, which are characterized by the loss of adipocyte markers, including FABP4. Brown adipocytes induced in WAT, also known as ‘‘beige’’ or ‘‘brite’’ cells [47], are derived from a distinct precursor population, separate from both mature white and brown adipocytes [48]. Several mechanisms have been proposed for WAT browning, including prolonged cold exposure, adrenergic activation, and the prostaglandin synthesis enzyme cyclooxygenase 2 (COX2) [49]. Additionally, previous studies indicated that a few cytokines or secreted proteins, such as AZGP1 [50], were associated with lipolysis and browning, ultimately leading to cancer cachexia [51]. These proteins also include IL1A, IL1B, IL6, IL10, TNFα [52], GDF15 [53], and PTHLH [9], most of which were highly expressed in tumors. Notably, AZGP1 was initially identified as a potential biomarker of prostate cancer [54], and its expression was subsequently shown to be closely associated with the histologic grade of breast cancer [55]. In addition, AZGP1 acted as a classical lipid mobilization factor and promoted the development of cancer cachexia. On the other hand, it has been pointed out that AZGP1 could inhibit EMT and cancer invasiveness by blocking the TGFβ-ERK pathway [56, 57]. Structurally, AZGP1 bears similarities to an MHC class I antigen-presenting molecule, suggesting it might also play a role in the immune response, but the underlying mechanism remains ambiguous. Therefore, whether AZGP1 functions as an oncogene or a tumor suppressor remains controversial, and it may play opposite roles in different tumor types.

Although AZGP1 expression was found to be elevated in both COAD and READ according to the TCGA Additional file 1: Fig. S2b), our study confirmed that MIIP did not affect AZGP1 expression (Fig. 3c) but instead regulated its secretion by altering the level of N-glycosylation. Consequently, the over-secreted AZGP1 acted as a mediator for tumor-surrounding WAT browning in a paracrine manner through the classical β-adrenergic receptor–cAMP–PKA pathway. Oligosaccharyl-transferase (OST) catalyses the transfer of a high-mannose glycan onto secretory proteins in the ER. In mammals, two distinct OST complexes are expressed, which function in a co-translational (OST-A, catalytic subunit STT3A) or post-translocational (OST-B, catalytic subunit STT3B) manner [58]. The STT3A isoform is primarily responsible for the co-translational glycosylation of the nascent polypeptide as it enters the lumen of the ER, while the STT3B isoform is required for efficient co-translational glycosylation of an acceptor site adjacent to the N-terminal signal sequence of a secreted protein [37]. Notably, our study confirmed that MIIP's interaction with AZGP1 more likely inhibits STT3A-mediated AZGP1 glycosylation but not STT3B (Fig. 3m and Additional file 1: Fig. S4f).

Adipocytes play a crucial role in the initiation and development of various tumors, promoting tumor metastasis, invasion, and poor prognosis. The interactions between adipocytes and tumors involve various metabolites, such as FFAs [41, 42], lactate [28], and creatine [59]. Tumors, including breast, gastric, colon, and ovarian cancers, often exhibit growth and undergo epithelial-mesenchymal transition (EMT) in the adipocyte-dominated tumor microenvironment (TME). These tumors rely on FFAs released from adipocytes and heavily depend on fatty acid oxidation (FAO) for ATP production, facilitated by pathways like the Ca^2+^/CaMKK-dependent AMPK or leptin-mediated JAK/STAT3 signaling [42, 60, 61]. This metabolic adaptation promotes their proliferation, metastasis, and other malignant behaviors, influencing processes such as autophagy, stemness, differentiation, and anoikis [8, 42, 62]. Our findings consistently demonstrate that FFAs secreted by peri-cancerous beige adipocytes, but not lactate, serve as the primary fuel for further CRC growth. FFAs not only fuel cancer progression through β-oxidation but also act as significant metabolic resources, generating abundant intermediate products within cells, such as lactate, Succ-CoA, Ac-CoA, and beta-hydroxybutyrate (BHB). These intermediates provide acyl groups to covalently modify proteins, thus regulating their activity, subcellular localization, or expression [63]. These modifications play vital roles in metabolomic-epigenetic and metabolomic-proteomic signaling pathways in tumors [63, 64]. Interestingly, the uptake of FFAs by cancer cells at low and high levels can have different effects—proliferative or lipotoxic, respectively. For instance, arachidonic acid promotes proliferation at low concentrations but induces lipotoxicity through ferroptosis at high concentrations. Furthermore, the uptake of small-chain versus very-long-chain FFAs may yield different impacts on cancer development [65]. Consequently, the nature of FFA uptake can have diverse effects and may be closely linked to various tumor types.

## Conclusions

In summary, our study elucidated the bidirectional communication between CRC and adjacent adipose tissue, which significantly contributes to CRC progression, highlighting the crucial regulatory role of MIIP in this process. Our data provided evidence of how reduced MIIP expression promotes white adipose tissue (WAT) browning by disrupting AZGP1 secretion through alterations in its N-glycosylation. Moreover, our findings underscored the tumor-supportive function of adjacent adipocytes in the tumor microenvironment (TME). Importantly, we demonstrated that the combination of chemotherapy drugs with inhibitors of WAT browning or FFAs uptake resulted in improved anti-cancer efficacy, presenting a novel therapeutic approach for treating CRC with abnormal MIIP expression.

## Methods

### Antibodies and reagents

The β-adrenergic receptor inhibitor (SR59230A, T13016), PKA inhibitors (H89-2HCl, T6250), FFA uptake inhibitor (Sulfosuccinimidyl oleate, T13036), MCT inhibitor (7ACC1, T5845), and N-linked glycosylation inhibitor (Tunicamycin, T13229) were procured from TopScienceBiochem (Shanghai, China). Oxaliplatin was obtained from MedChemExpress (HY-17371, Monmouth Junction, NJ). PNGase F (P0704S) and O-glycosidase (P0733S) were purchased from New England BioLabs (Ipswich, MA, US). For in vitro experiments, each chemical was dissolved in dimethyl sulfoxide (DMSO) and diluted to their indicated concentrations with the medium (e.g., final concentration, 20 μM H89-2HCl, 1 μM SR59230A). In the in vivo experiments, each chemical was dissolved in a vehicle consisting of 5% DMSO (Sigma), 7% dimethyl-acetoacetamide (DMA) (Sigma), and 10% Cremophor EI (Sigma), and 77% corn oil (Sigma). The AZGP1 ELISA kit was purchased from RayBiotech Life, Inc. (EIA-ZAG, Peachtree Corners, GA, US), the TNFα (EHC103a.96), and IL-6 ELISA kit (EHC007.96) were purchased from NeoBioscience (Shenzhen, Guangdong, China). The ER and Golgi apparatus protein enrichment kit (EX1260, EX1240) were obtained from Solarbio Life Sciences (Beijing, China). ER-Tracker Green (C1042S), Anti-Flag Magnetic Beads (P2115), Anti-His Magnetic Beads (P2135), and Mouse IgG Magnetic Beads (P2171) were purchased from Beyotime Biotechnology (Shanghai, China).

The primary antibodies used in this study included anti-UCP1 (23,673–1-AP, RRID: AB_2828003), anti-PGC-1α (66,369–1-Ig, RRID: AB_2828002), anti-FABP4 (12,802–1-AP, RRID: AB_2102442), anti-STT3A (12,034–1-AP, RRID: AB_2877818), and anti-STT3B (15,323–1-AP, RRID: AB_2198046), which were purchased from Proteintech Group, Inc (Rosemont, IL, US). Additionally, we used anti-phospho-(Ser/Thr) PKA Substrate (#9624S, RRID: AB_331817), anti-Perilipin1 (#9349, RRID: AB_10829911), anti-STAT3 (#12,640, RRID: AB_2629499), anti-phospho-STAT3 (Ser727)(#9136, RRID: AB_331755), anti-P65(#8242, RRID: AB_10859369), anti-phospho-P65 (Ser536)(#3036, RRID: AB_331281), anti-ERK1/2 (#4695, RRID: AB_390779), anti-phospho-ERK1/2 (Tyr204)(#9106, RRID: AB_331768), anti-PARP (#9532, RRID: AB_659884), anti-Caspase3 (#9662, RRID: AB_331439), anti-DYKDDDDK tag (#8146, RRID: AB_10950495), anti-α-tubulin (#2144, RRID: AB_2210548), and anti-GAPDH (#5174, RRID: AB_10622025) from Cell Signaling Technology (Danvers, MA, US). Furthermore, we used anti-CD36 (ab252923), anti-GRP94 (ab238126), anti-TGN46 (ab271183), anti-6 × His tag (ab18184), and anti-Ki67 (ab16667) from Abcam (Cambridge, UK). For other antibodies, we used MIIP (PA5-100,572, RRID: AB_2850081) and AZGP1 (PA5-76,728) from ThermoFisher Scientific Inc. (Waltham, MA, US), and anti-phospho-HSL (Ser660) (#AF8026, RRID: AB_2840089) and anti-total HSL (#AF6403, RRID: AB_2835234) from Affinity Biosciences (Cincinnati, OH, US). Additionally, we used CD105-PE (800,503, RRID: AB_2629654), CD34-FITC (343,503, RRID: AB_1731923), and CD45-FITC (304,005, RRID: AB_314393) from BioLegend (San Diego, California, US). Lastly, anti-β-Actin (bs-0061R, RRID: AB_10855480) was purchased from Bioss Inc. (Boston, MA, US).

### Plasmid construction and lentivirus preparation

The vector pCMV-MIIP-Flag was previously constructed in a study [24]. Furthermore, the MIIP CDS was amplified and cloned into the pcDNA3.1( +) vector (Thermo Scientific, Waltham, MA) with the addition of Streptavidin tag (S) and Flag tag (F). We developed AZGP1-6 × His NQ mutants (N109Q, N112Q, N128Q, N259Q), 2NQ (N128Q/N259Q), and 3NQ (N112Q/N128Q/N259Q) through site-directed mutagenesis using the pEX-3 (CMV-Neo) expression vector (GenePharma, Shanghai, China).

For the knockdown of human CD36, we transduced CD36 or control shRNA constructs (Origene, TR314090) into parental HCT116 cells following the manufacturer's instructions. To knock down AZGP1, we used commercial siRNAs (sc-36865, Santa Cruz Biotechnology, Santa Cruz, CA, USA) that are pools of three target-specific 20-25nt siRNAs designed to knock down gene expression.

Regarding mouse *Miip* overexpression, we amplified *Miip* CDS by PCR and cloned it into the LV17 (EF-1α-Luciferase-puro) shuttle vector (GenePharma, Shanghai, China) using Not I and Bam HI restriction enzymes (New England Biolabs, Ipswich, MA). For specific knockdown of mouse *Miip*, we constructed lentiviral-based shRNA constructs in the LV16 (U6-Luciferase-puro) shuttle vector (GenePharma, Shanghai, China) against *Miip*, and confirmed the correctness of the resulting construct by DNA sequencing. We obtained stable overexpression cell lines: HCT116-MIIP, CT26.WT-Miip, and CMT93-Miip, as well as a stable knockdown cell line: HCT116-shCD36 #1-#3, CT26.WT-shMiip #1-#2, and CMT93-shMiip #1-#2, through lentivirus infection and puromycin selection. The shRNA oligonucleotides specific for Miip (Miip shRNA) are listed in Additional file 2: Table S4.

### Patients’ samples

Tissue microarrays (TMAs) containing 172 cases of colorectal cancer patients (148 cases of adenocarcinomas and 24 cases of mucinous adenocarcinoma), along with 47 cases of matched adjacent normal tissue, were obtained from Avilabio Technology Co., LTD, Xi’an, Shaanxi. Additionally, we collected freshly dissected colorectal cancer and adjacent normal tissues from 14 patients with colorectal cancer, as well as 9 cases of paraffin-embedded colorectal cancer tissue with varying grades, from Xijing Digestive Disease Hospital. Clinicopathological characteristics of CRC patients are summarized in Additional file 2: Table S5. All samples underwent histologic evaluation by pathologists and were diagnosed according to the World Health Organization classification (WHO Fourth Edition published in 2016). Moreover, freshly dissected abdominal and inguinal subcutaneous fat tissues were obtained from the Department of Burn and Skin Surgery of Xijing Hospital. All samples were collected with the informed consent of the patients, and the experiments were approved by the Research Ethics Committee (No.KY20180403-1), Xijing Hospital, Fourth Military Medical University (Shaanxi, Xian, China), and adhered to the principles set out in the WMA Declaration of Helsinki and the Department of Health and Human Services Belmont Report.

### Cell lines and primary white adipocyte culture

HEK-293 cells (RRID: CVCL_0045), human colon cancer cell lines HCT116 (RRID: CVCL_0291) and HT29 (RRID: CVCL_0320), as well as mouse colorectal cancer cell lines CT26.WT (RRID: CVCL_7256) and CMT93 (RRID: CVCL_1986), and mouse pre-adipocytes 3T3-L1 (RRID: CVCL_0123), were all obtained from ATCC, US. HCT116 cells with MIIP haploinsufficiency, generated using zinc finger nuclease technology, were constructed in a previous study [19] and generously provided by Dr. Wei Zhang (Wake Forest Baptist Medical Center, Winston-Salem, NC). All these cells were routinely maintained in Dulbecco's Modified Eagle Medium (DMEM), McCoy’s 5A or RPMI-1640 medium (Life Technologies, US), respectively, supplemented with 10% fetal bovine serum, and cultured at 37 °C in a humidified atmosphere comprising 5% CO_2_.

For the isolation of human adipose-derived stem cells (hADSCs), we dissected abdominal and inguinal fat tissue, washed it with PBS, minced it, and subjected it to digestion for 1 h at 37 °C in PBS containing α-MEM medium (Gibco) and 1.5 mg/mL collagenase I (Roche). The digested tissue was then filtered through a 200-μm cell strainer and centrifuged at 300 g for 5 min to pellet the ADSCs. Subsequently, the cells were resuspended in adipocyte culture medium (α-MEM medium supplemented with 2% glutaMax, 1% pen/strep, and 5% PLTMax), centrifuged as before, and then plated. The ADSCs were allowed to grow to confluency for adipocyte differentiation. This differentiation process was induced by using an adipogenic cocktail comprising 1 μM dexamethasone, 10 μg/mL insulin, 0.5 mM isobutylmethylxanthine (IBMX), and 10 μM rosiglitazone in adipocyte culture medium A for 2 days. Subsequently, the cells were maintained in adipocyte culture medium B, which only contained 10 μg/mL insulin, for another 1 day. Regarding the 3T3-L1 cells, the process to mature them into beige cells was almost identical to that of ADSCs, with the exception of the adipogenic cocktail formulation used, which consisted of 0.25 μM dexamethasone, 1 μg/mL insulin, 0.5 mM IBMX, and 2 μM rosiglitazone.

### Mice and mice housing

Male BALB/c, C57BL/6, and nude mice, aged 4 to 6 weeks, were procured from Beijing GemPharmatech for the study. The sample size was determined using the "resource equation" method [66], and we utilized the power analysis tool PASS v16 (Utah, USA, 2018) to estimate the minimum detectable effect size for the selected sample size. Given the multiple cell groups involved in the animal experiment, we required a minimum of 110 mice. Considering potential attrition or natural deaths, a total of 120 mice were used in this study. All mice were group-housed under specific pathogen-free conditions, with a constant temperature (22–25 °C), humidity (60%), and a 12-h light/dark cycle. They were provided with ad libitum access to food and water. The mice were allowed to acclimatize for 1 or 2 weeks after arrival at the facility before being used in the experiments. Throughout the study, the mice displayed normal health. Ethical approval for all procedures involving animals was obtained from the Institutional Animal Care and Use Committee of Fourth Military Medical University (No.20180315).

For subcutaneous xenograft studies, HCT116 cells (1 × 10^6^ cells) were mixed with PBS or differentiated mature ADSC cells (2.5 × 10^5^ cells). For subcutaneous allograft studies, CT26.WT cells or CMT93 cells (2 × 10^5^ cells) were mixed with PBS or differentiated mature 3T3-L1 cells (0.5 × 10^5^ cells). These cells were combined at a ratio of 4:1 with the adipogenic cocktail and implanted subcutaneously into the right flank of the mice along with Matrigel in 100μL (Corning, #354,248). Tumor growth was monitored by measuring the tumor diameter every 2 or 3 days using a digital caliper. The tumor volumes were calculated as 0.5 × (longest diameter) × (shortest diameter) × (shortest diameter).

For bioluminescence imaging and analysis, mice were anesthetized with 3% isoflurane every 7 days to monitor tumor status. D-Luciferin (Xenogen) was injected at a dose of 150 mg/kg (body weight). Five minutes later, bioluminescent images were acquired using an IVIS imaging system (Xenogen). Analysis was performed using LivingImage software (Xenogen) by measuring the photon flux within a region of interest drawn around the bioluminescence signals. Blank regions of interest were also measured for each scan and deducted from each tumor photon flux for normalization. At the end of the experiments, tumors dissected from individual mice were weighed, fixed in 4% paraformaldehyde, or flash frozen under liquid nitrogen.

### Quantitative real-time PCR analysis

Total RNA was extracted using RNAiso reagent (TaKaRa, Dalian, China) following the manufacturer’s protocol. Subsequently, the first strand cDNA was synthesized from 2 μg of total RNA using reverse transcriptase, either for coding region genes or non-coding regions, and utilized as the template for RT-qPCR analysis. For this analysis, cDNA from *ACTB* (*Homo sapiens*) or *Actb* (*Mus musculus*) was employed as internal controls. The primers used in the PCR process are listed in Additional file 2: Table S6. The PCR was carried out using a GeneAmp PCR system 2400 Thermal Cycler (Perkin-Elmer, Norwalk CT, US). The PCR temperature program consisted of 40 cycles, each comprising 30 s at 95 °C, 10 s at 95 °C, and 30 s at 60 °C.

### Statistical analysis

The results were analyzed using SPSS 19.0 software and GraphPad Prism 8 software. All experiments were conducted at least three times. Quantitative data are presented as the mean ± standard deviation (SD). For the comparison of two groups, Student's t-test (parametric test) was performed for continuous variables with a normal distribution, while the Mann–Whitney test (non-parametric test) was used for data with a skewed distribution. In the case of multiple comparisons, the ANOVA test (parametric test) followed by the Bonferroni test was employed for data with a normal distribution, and the Kruskal–Wallis test (non-parametric test) was used for data with a skewed distribution. Bonferroni correction was applied to adjust the P value in multiple comparisons. Survival curves were plotted using the Kaplan–Meier method, and the survival rates were compared using the log-rank test. Statistical significance was denoted as *P < 0.05, **P < 0.01, and ***P < 0.001.

More detailed experimental procedures can be found in Additional file 3.

### Supplementary Information

**Additional file 1**: Supplementary Figures.

**Additional file 2**: Supplementary Tables.

**Additional file 3**: Supplementary methods and supplementary figure legends.

## Data Availability

The data that support the findings of this study are available from the corresponding author upon reasonable request.

## Abbreviations

MIIP: Migration and invasion inhibitory protein
CRC: Colorectal cancer
WAT: White adipose tissue
TME: Tumor microenvironment
FFA: Free fatty acid
AZGP1: Alpha-2-glycoprotein 1, zinc-binding

## References

[1] Schmitt M, Greten FR (2021). The inflammatory pathogenesis of colorectal cancer. Nat Rev Immunol.

[2] Ulrich CM, Himbert C, Holowatyj AN, Hursting SD (2018). Energy balance and gastrointestinal cancer: risk, interventions, outcomes and mechanisms. Nat Rev Gastroenterol Hepatol.

[3] Tabuso M, Homer-Vanniasinkam S, Adya R, Arasaradnam RP (2017). Role of tissue microenvironment resident adipocytes in colon cancer. World J Gastroenterol.

[4] Park J, Morley TS, Kim M, Clegg DJ, Scherer PE (2014). Obesity and cancer–mechanisms underlying tumour progression and recurrence. Nat Rev Endocrinol.

[5] Ko JH, Um JY, Lee SG, Yang WM, Sethi G, Ahn KS (2019). Conditioned media from adipocytes promote proliferation, migration, and invasion in melanoma and colorectal cancer cells. J Cell Physiol.

[6] Di Franco S, Bianca P, Sardina DS, Turdo A, Gaggianesi M, Veschi V (2021). Adipose stem cell niche reprograms the colorectal cancer stem cell metastatic machinery. Nat Commun.

[7] Zhang Q, Deng T, Zhang H, Zuo D, Zhu Q, Bai M (2022). Adipocyte-derived exosomal MTTP suppresses ferroptosis and promotes chemoresistance in colorectal cancer. Adv Sci.

[8] Xiong X, Wen YA, Fairchild R, Zaytseva YY, Weiss HL, Evers BM (2020). Upregulation of CPT1A is essential for the tumor-promoting effect of adipocytes in colon cancer. Cell Death Dis.

[9] Kir S, White JP, Kleiner S, Kazak L, Cohen P, Baracos VE (2014). Tumour-derived PTH-related protein triggers adipose tissue browning and cancer cachexia. Nature.

[10] Liu P, Huang S, Ling S, Xu S, Wang F, Zhang W (2019). Foxp1 controls brown/beige adipocyte differentiation and thermogenesis through regulating β3-AR desensitization. Nat Commun.

[11] Ahmadian M, Abbott MJ, Tang T, Hudak CS, Kim Y, Bruss M (2011). Desnutrin/ATGL is regulated by AMPK and is required for a brown adipose phenotype. Cell Metab.

[12] Petruzzelli M, Schweiger M, Schreiber R, Campos-Olivas R, Tsoli M, Allen J (2014). A switch from white to brown fat increases energy expenditure in cancer-associated cachexia. Cell Metab.

[13] Morak M, Schmidinger H, Riesenhuber G, Rechberger GN, Kollroser M, Haemmerle G (2012). Adipose triglyceride lipase (ATGL) and hormone-sensitive lipase (HSL) deficiencies affect expression of lipolytic activities in mouse adipose tissues. Mol Cell Proteomics.

[14] Agustsson T, Rydén M, Hoffstedt J, van Harmelen V, Dicker A, Laurencikiene J (2007). Mechanism of increased lipolysis in cancer cachexia. Can Res.

[15] Thompson MP, Cooper ST, Parry BR, Tuckey JA (1993). Increased expression of the mRNA for hormone-sensitive lipase in adipose tissue of cancer patients. Biochem Biophys Acta.

[16] Nieman KM, Romero IL, Van Houten B, Lengyel E (2013). Adipose tissue and adipocytes support tumorigenesis and metastasis. Biochem Biophys Acta.

[17] Weber BZC, Arabaci DH, Kir S (2022). Metabolic reprogramming in adipose tissue during cancer cachexia. Front Oncol.

[18] Song SW, Fuller GN, Khan A, Kong S, Shen W, Taylor E (2003). IIp45, an insulin-like growth factor binding protein 2 (IGFBP-2) binding protein, antagonizes IGFBP-2 stimulation of glioma cell invasion. Proc Natl Acad Sci USA.

[19] Sun Y, Ji P, Chen T, Zhou X, Yang D, Guo Y (2017). MIIP haploinsufficiency induces chromosomal instability and promotes tumour progression in colorectal cancer. J Pathol.

[20] Sun D, Wang Y, Jiang S, Wang G, Xin Y (2018). MIIP is downregulated in gastric cancer and its forced expression inhibits proliferation and invasion of gastric cancer cells in vitro and in vivo. Onco Targets Ther.

[21] Fang J, Chen YL, Yao HB, Peng SS, Yang P, Ding ZY (2020). MIIP inhibits malignant progression of hepatocellular carcinoma through regulating AKT. Eur Rev Med Pharmacol Sci.

[22] Yan G, Ru Y, Yan F, Xiong X, Hu W, Pan T (2019). MIIP inhibits the growth of prostate cancer via interaction with PP1α and negative modulation of AKT signaling. Cell Commun Signal.

[23] Hu W, Yan F, Ru Y, Xia M, Yan G, Zhang M (2020). MIIP inhibits EMT and cell invasion in prostate cancer through miR-181a/b-5p-KLF17 axis. Am J Cancer Res.

[24] Yan F, Wang Q, Xia M, Ru Y, Hu W, Yan G (2021). MIIP inhibits clear cell renal cell carcinoma proliferation and angiogenesis via negative modulation of the HIF-2α-CYR61 axis. Cancer Biol Med.

[25] Yang F, Duan M, Zheng F, Yu L, Wang Y, Wang G (2021). Fas signaling in adipocytes promotes low-grade inflammation and lung metastasis of colorectal cancer through interaction with Bmx. Cancer Lett.

[26] Kir S, Spiegelman BM (2016). Cachexia & brown fat: a burning issue in cancer. Trends in cancer.

[27] Paré M, Darini CY, Yao X, Chignon-Sicard B, Rekima S, Lachambre S (2020). Breast cancer mammospheres secrete Adrenomedullin to induce lipolysis and browning of adjacent adipocytes. BMC Cancer.

[28] Wei G, Sun H, Dong K, Hu L, Wang Q, Zhuang Q (2021). The thermogenic activity of adjacent adipocytes fuels the progression of ccRCC and compromises anti-tumor therapeutic efficacy. Cell Metab.

[29] Egan JJ, Greenberg AS, Chang MK, Londos C (1990). Control of endogenous phosphorylation of the major cAMP-dependent protein kinase substrate in adipocytes by insulin and beta-adrenergic stimulation. J Biol Chem.

[30] Mutoh M, Teraoka N, Takasu S, Takahashi M, Onuma K, Yamamoto M (2011). Loss of adiponectin promotes intestinal carcinogenesis in Min and wild-type mice. Gastroenterology.

[31] Endo H, Hosono K, Uchiyama T, Sakai E, Sugiyama M, Takahashi H (2011). Leptin acts as a growth factor for colorectal tumours at stages subsequent to tumour initiation in murine colon carcinogenesis. Gut.

[32] Huang XY, Huang ZL, Yang JH, Xu YH, Sun JS, Zheng Q (2016). Pancreatic cancer cell-derived IGFBP-3 contributes to muscle wasting. J Exp Clin Cancer Res.

[33] Bing C, Bao Y, Jenkins J, Sanders P, Manieri M, Cinti S (2004). Zinc-alpha2-glycoprotein, a lipid mobilizing factor, is expressed in adipocytes and is up-regulated in mice with cancer cachexia. Proc Natl Acad Sci USA.

[34] Hassan MI, Waheed A, Yadav S, Singh TP, Ahmad F (2008). Zinc alpha 2-glycoprotein: a multidisciplinary protein. Mol Cancer Res.

[35] Tisdale MJ (2009). Zinc-alpha2-glycoprotein in cachexia and obesity. Curr Opin Support Palliat Care.

[36] Romauch M (2020). Zinc-α2-glycoprotein as an inhibitor of amine oxidase copper-containing 3. Open Biol.

[37] Ruiz-Canada C, Kelleher DJ, Gilmore R (2009). Cotranslational and posttranslational N-glycosylation of polypeptides by distinct mammalian OST isoforms. Cell.

[38] Cherepanova N, Shrimal S, Gilmore R (2016). N-linked glycosylation and homeostasis of the endoplasmic reticulum. Curr Opin Cell Biol.

[39] Nieman KM, Kenny HA, Penicka CV, Ladanyi A, Buell-Gutbrod R, Zillhardt MR (2011). Adipocytes promote ovarian cancer metastasis and provide energy for rapid tumor growth. Nat Med.

[40] Wang YY, Attané C, Milhas D, Dirat B, Dauvillier S, Guerard A (2017). Mammary adipocytes stimulate breast cancer invasion through metabolic remodeling of tumor cells. JCI insight.

[41] Zhang M, Di Martino JS, Bowman RL, Campbell NR, Baksh SC, Simon-Vermot T (2018). Adipocyte-derived lipids mediate melanoma progression via FATP proteins. Cancer Discov.

[42] Wen YA, Xing X, Harris JW, Zaytseva YY, Mitov MI, Napier DL (2017). Adipocytes activate mitochondrial fatty acid oxidation and autophagy to promote tumor growth in colon cancer. Cell Death Dis.

[43] Tian W, Zhang W, Zhang Y, Zhu T, Hua Y, Li H (2020). FABP4 promotes invasion and metastasis of colon cancer by regulating fatty acid transport. Cancer Cell Int.

[44] Mukherjee A, Bilecz AJ, Lengyel E (2022). The adipocyte microenvironment and cancer. Cancer Metastasis Rev.

[45] Bruno G, Cencetti F, Pini A, Tondo A, Cuzzubbo D, Fontani F (2020). β3-adrenoreceptor blockade reduces tumor growth and increases neuronal differentiation in neuroblastoma via SK2/S1P(2) modulation. Oncogene.

[46] Drury J, Rychahou PG, He D, Jafari N, Wang C, Lee EY (2020). Inhibition of fatty acid synthase upregulates expression of cd36 to sustain proliferation of colorectal cancer cells. Front Oncol.

[47] Harms M, Seale P (2013). Brown and beige fat: development, function and therapeutic potential. Nat Med.

[48] Wang QA, Tao C, Gupta RK, Scherer PE (2013). Tracking adipogenesis during white adipose tissue development, expansion and regeneration. Nat Med.

[49] Villarroya F, Vidal-Puig A (2013). Beyond the sympathetic tone: the new brown fat activators. Cell Metab.

[50] Mracek T, Stephens NA, Gao D, Bao Y, Ross JA, Rydén M (2011). Enhanced ZAG production by subcutaneous adipose tissue is linked to weight loss in gastrointestinal cancer patients. Br J Cancer.

[51] Kasprzak A (2021). The role of tumor microenvironment cells in colorectal cancer (CRC) cachexia. Int J Mol Sci..

[52] Argilés JM, Busquets S, López-Soriano FJ (2003). Cytokines in the pathogenesis of cancer cachexia. Curr Opin Clin Nutr Metab Care.

[53] Tsai VW, Husaini Y, Manandhar R, Lee-Ng KK, Zhang HP, Harriott K (2012). Anorexia/cachexia of chronic diseases: a role for the TGF-β family cytokine MIC-1/GDF15. J Cachexia Sarcopenia Muscle.

[54] Frenette G, Dubé JY, Lazure C, Paradis G, Chrétien M, Tremblay RR (1987). The major 40-kDa glycoprotein in human prostatic fluid is identical to Zn-alpha 2-glycoprotein. Prostate.

[55] Díez-Itza I, Sánchez LM, Allende MT, Vizoso F, Ruibal A, López-Otín C (1993). Zn-alpha 2-glycoprotein levels in breast cancer cytosols and correlation with clinical, histological and biochemical parameters. Eur J Cancer.

[56] Kong B, Michalski CW, Hong X, Valkovskaya N, Rieder S, Abiatari I (2010). AZGP1 is a tumor suppressor in pancreatic cancer inducing mesenchymal-to-epithelial transdifferentiation by inhibiting TGF-β-mediated ERK signaling. Oncogene.

[57] Xu MY, Chen R, Yu JX, Liu T, Qu Y, Lu LG (2016). AZGP1 suppresses epithelial-to-mesenchymal transition and hepatic carcinogenesis by blocking TGFβ1-ERK2 pathways. Cancer Lett.

[58] Ramírez AS, Kowal J, Locher KP (2019). Cryo-electron microscopy structures of human oligosaccharyltransferase complexes OST-A and OST-B. Science.

[59] Maguire OA, Ackerman SE, Szwed SK, Maganti AV, Marchildon F, Huang X (2021). Creatine-mediated crosstalk between adipocytes and cancer cells regulates obesity-driven breast cancer. Cell Metab.

[60] Martinez-Outschoorn UE, Sotgia F, Lisanti MP (2012). Power surge: supporting cells "fuel" cancer cell mitochondria. Cell Metab.

[61] Snaebjornsson MT, Janaki-Raman S, Schulze A (2020). Greasing the Wheels of the cancer machine: the role of lipid metabolism in cancer. Cell Metab.

[62] Wang YN, Zeng ZL, Lu J, Wang Y, Liu ZX, He MM (2018). CPT1A-mediated fatty acid oxidation promotes colorectal cancer cell metastasis by inhibiting anoikis. Oncogene.

[63] Sabari BR, Zhang D, Allis CD, Zhao Y (2017). Metabolic regulation of gene expression through histone acylations. Nat Rev Mol Cell Biol.

[64] Fu Y, Yu J, Li F, Ge S (2022). Oncometabolites drive tumorigenesis by enhancing protein acylation: from chromosomal remodelling to nonhistone modification. J Exp Clin Cancer Res.

[65] Panaroni C, Fulzele K, Mori T, Siu KT, Onyewadume C, Maebius A (2022). Multiple myeloma cells induce lipolysis in adipocytes and uptake fatty acids through fatty acid transporter proteins. Blood.

[66] Charan J, Kantharia ND (2013). How to calculate sample size in animal studies?. J Pharmacol Pharmacother.

