# Supplementary material for: MIIP downregulation drives colorectal cancer progression through inducing peri-cancerous adipose tissue browning
Source: Cell Biosci. 2024 Jan 20;14:12. doi: 10.1186/s13578-023-01179-0 (PMC10800076; doi:10.1186/s13578-023-01179-0)
Supplement: Supplementary file 3 — Additional file 3: Supplementary methods and supplementary figure legends. [file 13578_2023_1179_MOESM3_ESM.docx]

**Supplementary Methods**

*Conditioned medium preparation*

For conditioned medium (CM) collection, HCT116, CT26.WT and CMT93 stable cell line were plated the day before and the media were changed to FreeStyle expression medium (12338026; Gibco, Thermo Fisher Scientific, US) next day. The CM were then collected 24 h later, centrifuged at 12 000g for 5 min, and filtered through a 0.22μm filter. Differentiated mature adipocytes were exposed to fresh media mixed with CM from cells above at a ratio of 1:1 (v/v) for 24h. For two-step CM transfer experiments, adipocytes were incubated with the conditioned medium derived from colorectal cancer cells for 24h, and later the supernatant were collected, centrifuged, and filtered as above, then mixed with fresh culture medium at 1:1 ratio culture medium and applied to treat human or mouse parental CRC cell lines (HCT116, HT29 or CMT93) for 24h for *in vitro* proliferation, Oil Red O staining and RT-qPCR.

*Cell Characterization*

Freshly isolated ADSCs were characterized by fluorescence-activated cell-sorting (FACS) analysis. The cells were incubated for 30 min at 4°C with fluorochrome-conjugated antibodies against the indicated antibodies or an isotype control. Antibodies used were: CD34-fluorescein isothiocyanate (FITC), CD45-FITC (pan-haematopoietic marker) and CD105-PE (mesenchymal marker; BioLegend, San Diego, California, US). Cells were washed and resuspended in FACS buffer (PBS, 0.5% human serum albumin, 0.5 mM EDTA) and analysed with a FACS Calibur flow cytometer (BD Biosciences US). The data were analysed using FlowJo software v. 10.0.6 (Tree Star Inc., Ashland, Oreg., US) and expressed as the percentage of positive cells determined by flow cytometry.

*RNA-seq and transcriptomic expression analysis*

HCT116-MIIP^+/-^ and WT control cells were collected and RNA was isolated for sequencing using the SMART-Seq™ v4 Ultra™ Low Input RNA Kit. PCR products were amplified and sequenced on an Illumina HiSeq™ 2500 platform by NovelBio (Shanghai, China). High-quality clean reads from all two samples were merged together and mapped to the reference sequence. To determine the biological significance of the differentially expressed genes, which were defined as genes with log2 expression fold change ≥ 0.5, or ≤ -0.5, functional classification and gene enrichment analysis were performed using GO Term (Biological Process level 5) with DAVID Bioinformatics Resources. Top ten highly enriched functional categories were listed and arranged in descending order of *p*-value of enrichment. Gene set enrichment analysis (GSEA) was launched to identify genes of statistical difference by using GSEA v3 software (http://www.brodinstitute.org/gsea/index.jsp).

*Tandem affinity purification and Mass Spectrometry analysis*

HEK-293T cells were transiently transfected with pcDNA3.1(+)-MIIP-SF or empty vector. After culturing for 48 hours, cells were lysed on ice in lysis buffer (30 mM Tris, pH 7.5, 150 mM NaCl, 1% Triton X-100). Protein lysates containing 2 mg total protein were precleaned and precipitated with anti-Streptavidin magnetic beads (Yeasen Biotechnology, Shanghai, China) for 6 h at 4°C, the beads were subsequently washed 3 times and the proteins were eluted and collected, followed by incubation with anti-FLAG magnetic beads (Beyotime Biotechnology, Shanghai, China) overnight at 4°C. The beads were then washed and the fractions were collected and resolved on SDS-polyacrylamide gel, silver stained, and subjected to LC-MS/MS sequencing and data analysis were performed by Applied Protein Technology Co., LTD (Shanghai, China).

*ER-Tracker staining and immunofluorescence assay*

5×10^3^ cells were plated on sterile glass coverslips, after 24 hours, the cells were washed and incubated with pre-warmed diluent containing 200 nM ER-Tracker Green probe (Beyotime) for 15-30 min and then washed twice, followed by fixation with 4% paraformaldehyde for 2-5 min at 37℃. The slips were then blocked with 3% BSA for 1 h at room temperature and then incubated with indicated antibodies at room temperature for 2 h. After washing with PBS, secondary antibody was incubated in the dark for 1 h at room temperature and then washed with PBS; the coverslips were counterstained with DAPI (0.1 mg/mL; Molecular Probes) and imaged with confocal microscope.

*Endoplasmic reticulum and Golgi apparatus protein enrichment*

Proteins of endoplasmic reticulum (ER) and Golgi apparatus fractions were prepared by ER and Golgi apparatus enrichment kit (EX1260, EX1240, Solarbio Life Sciences, Beijing, China), respectively. All experiments were performed according to per manufacturer’s instruction. Protein concentration was determined using the BCA protein assay kit (P0012, Beyotime Biotechnology, Shanghai, China). Proteins from each fraction was analyzed by immunoblotting analysis using primary antibodies against GRP94 and TGN46, then subjected to Co-IP assay.

*Seahorse XF-24 measurements*

The Seahorse Bioscience XF-24 Flux Analyzer (Agilent Technologies) was used to measure the OCR of tumor cells according to the manufacturer’s protocol (Calton et al., 2016). In brief, HCT116 cells were seeded at a density of 3×10^4^ cells/well into 24-well plates and allowed for adherent to the bottom overnight, followed by treatment with the corresponding CM (McCoy's 5A or MIIP^+/+^ CM→Adipocytes or MIIP^+/-^ CM→Adipocytes) for additional 24 h. OCR were measured under basal condition for 4 cycles, and inhibitors were sequentially added at the indicated time points: Etomoxir (50 μM), antimycin A (0.2 μM) and rotenone (0.2 μM), then OCR was automatically calculated by the WAVE software (Agilent). All parameters were normalized to total protein amount in individual wells using the BCA protein assay (Beyotime Biotechnology).

*Immunoblots*

Total protein was extracted either from cultured cells or tumor samples using RIPA buffer and quantified using a commercial BCA kit (Beyotime Biotechnology, Shanghai, China). The protein samples were resolved by SDS-PAGE on 8 to 15% polyacrylamide gels and transferred to nitrocellulose membranes. The membranes were blocked and then probed with the indicated primary antibodies and corresponding secondary antibodies, and washed with TBST buffer (PH 8.0), then developed using the enhanced chemiluminescence kit (Tanon, Shanghai, China).

*Co-immunoprecipitation (Co-IP)*

Cells were transiently transfected with indicated plasmids. After culturing for 48 hours, cells were lysed on ice in lysis buffer (30 mM Tris, pH 7.5, 150 mM NaCl, 1% Triton X-100). Protein lysates containing 1 mg total protein were precleaned and precipitated with indicated antibody or IgG (Cell Signaling Technology, Danvers, MA, US) for 6 h at 4°C, followed by incubation with protein A+G sepharose IP beads (Santa Cruz Biotechnology, CA, US) overnight at 4°C. IP beads were subsequently washed 3 times with lysis buffer and boiled in SDS sample buffer for 10 min. Samples were then separated by SDS-PAGE followed by immunoblot with indicated antibodies.

*Cell proliferation assay*

Cells were seeded into 96-well plates in septuplets at 1×10^3^ per well, and cell viability was tested by a CCK-8 Kit (Dojindo, Japan) every 24 h. The absorbance of each well was measured at 450 nm using a microplate reader (Bio-Rad, US). The data were presented as the mean ± SD.

*Cell invasion assays*

Cell invasion assays were performed in matrigel-coated transwell chambers (8-μm pore size, BD Pharmingen, US). Cells in 0.1% FBS medium were seeded at 2×10^4^ per upper chamber, and then placed into 24-well tissue culture plates containing 10% FBS medium. After 24h, invasive cells were stained with Giemsa and analyzed with microscope. The number of invading cells was determined by counting ten high-power fields (×400) on each membrane and calculated as the mean number of cells per field.

*Flow cytometry analysis of apoptosis*

HCT116 and CMT93 cells were incubated with different indicated medium for 24 h, and then washed and medium was changed. After another 24 h, oxaliplatin was added, and cells were treated for 24 h. The apoptotic cells were evaluated by propidium iodine and Annexin V-FITC staining (BD, USA) and analyzed with FACScan apparatus. Early apoptotic cells were defined as PI-negative, Annexin V-positive cells. The data were presented as the mean ± SD.

*Enzyme-linked immunosorbent assay (ELISA)*

The concentration of the AZGP1, TNFα and IL-6 protein released in the supernatant was tested by commercial ELISA kits (RayBiotech, US). Cells were seeded into 6-well plates at 2×10^5^ per well and cultured in 2 mL medium for 24 h. Then, the culture supernatants were harvested and transferred into a 96-well ELISA plate (100 μL per well) and incubated at 37 °C for 90 min. Afterward, the supernatants were aspirated, and the plate was incubated with specific antibodies for another 60 min at 37 °C, followed by incubation with ABC solution within 30 min. The TMB solution was added to the well and incubated in the dark for 20 min, and the optimal density reading was determined by a microplate reader at 450 nm.

*Histological evaluation and immunohistochemistry (IHC)*

For histological examination, tissues as indicated were harvested, fixed with 4% paraformaldehyde in PBS, embedded into paraffin blocks, sectioned, and then stained with H&E (Sigma) following standard protocol. Bright-field images were acquired using microscope (Olympus, Japan). For immunohistochemistry (IHC) analysis, deparaffinized sections were incubated with indicated primary antibody at 4℃ overnight, and then incubated species-appropriate secondary antibodies. The images were obtained with inverted microscope (Olympus, Japan) and digital slice scanner (3DHISTECH, Hungary). The staining score was calculated by multiplying the stained area (%) score (≤5%: 0, 6-25%: 1, 25-50%: 2, 51-75%: 3, ≥75%: 4) and the intensity score (colorless: 0, mild: 1, moderate: 2, strong: 3). High expression was specified as a score greater than or equal to 9, while low expression corresponded to a score less than 9. All scoring work was performed independently by two pathologists.

*Secretory protein profile analysis*

HCT116-MIIP^+/-^ and WT control cells were cultured with serum-free media for 24 h. The conditioned medium samples were collected and centrifuged at 3 000 × g for 5 min to remove dead cells and debris, and followed by immediately snap frozen. Liquid chromatography-tandem mass spectrometry (LC-MS/MS) analysis were performed by BGI Technology Co., LTD (Shenzhen, Guangdong, China).

*Determination of FFAs and glycerin concentration*

Fully differentiated mature adipocytes were exposed to fresh media mixed with CM from colorectal cancer cells at a ratio of 1:1 (v/v) for 24h. Then the cellular supernatants were collected, and the FFA and glycerin concentration was measured using a commercial colorimetric kit (Nanjing Jiancheng Bioengineering Institute, Nanjing, China) according to the manufacturer's instructions.

*Oil Red O staining*

Cells were washed twice with PBS and fixed with formaldehyde at room temperature for 20 minutes. Then 0.5% Oil Red O was added and incubate for 1 hour, followed by washing with 70% ethanol solution. Finally, an inverted microscope was used to observe and record photographs.

**Supplementary Figure Legends**

**Supplementary Figure 1.** Identification and differentiation of primary adipose-derived stem cells (ADSCs). **(a)** MIIP expression levels in the TMAs shown in (Fig.1C) were quantified with scoring system. The staining score was calculated by multiplying the stained area (%) score and the intensity score, and the correlation between MIIP expression and colorectal cancer grade was statistically analyzed. Expression group: low, score < 9; high, score ≥ 9. **(b)** Immunoblots analysis of MIIP protein expression in HCT116 cells with *MIIP* haploinsufficiency. **(c)** Representative images of isolated primary ADSCs identified by flow cytometry (3 biological replicates). **(d)** Representative images of Oil Red O staining of ADSCs and differentiated mature adipocytes (40× for images with a 50 μm scale bar, 3 biological replicates).

**Supplementary Figure 2.** MIIP binds with AZGP1. **(a)** Volcano plots of secreted proteins with significantly changed abundance in MIIP^+/-^ cell supernatants compared to MIIP^+/+^ cell supernatants. (Adjusted *P* < 0.05, fold change > 1.3. Red: upregulated; Green: downregulated; Grey: unchanged. 3 biological replicates per group). **(b)** mRNA levels of AZGP1 in COAD, READ and corresponding normal tissues analyzed from The Cancer Genome Atlas (TCGA) data sets, ^*^*P* < 0.05. **(c)** *Miip* mRNA level was detected in stable *Miip* knockdown CT26.WT and CMT93 cells by RT-qPCR (3 biological replicates, all data are shown as mean ± SD. ^***^*P* < 0.001). **(d)** Tandem affinity purification of MIIP-containing protein complexes. Cellular extracts from HEK-293T cells expressing SFB tag (control) or MIIP-SFB were immunopurified with anti-Streptavidin and anti-Flag magnetic beads successively, and then eluted. Eluates were analyzed by SDS-PAGE. Protein bands were excised and subjected to MS analysis. S: Streptavidin, F: Flag. **(e-f)** Co-immunoprecipitation analysis of MIIP and AZGP1 in HepG2 (e) and T47-D (f) cells transfected with pCMV-MIIP-Flag, by immunoprecipitation with anti-Flag and immunoblot with anti-AZGP1. **(g)** Immunoblot analysis was performed to examine the specific siRNA-induced *AZGP1* knockdown in HCT116-MIIP^+/-^ cells.

**Supplementary Figure 3.** MIIP affects the N-glycosylation and secretion of AZGP1. **(a)** Glycosylation pattern of AZGP1 protein in HCT116 control and MIIP-overexpressing cells. Cell lysates were treated with O-glycosidase and analyzed by immunoblots. **(b)** The concentration of AZGP1 was determined by ELISA in the supernatant of HCT116 cells treated as described in (Fig.3h) (7 biological replicates per group, ^*^*P* < 0.05, ^***^*P*<0.001). **(c)** The extracted ER and Golgi protein samples were identified with ER marker GRP94 and Golgi marker TGN46 by immunoblot, respectively. **(d)** HCT116 and HepG2 cells were subjected to ER-Tracker staining combined with immunofluoresence assay using anti-MIIP and anti-AZGP1. Scale bar, 10 μm. **(e-f)** Prediction of potential N-glycosylation modification sites of AZGP1 using NetNGlyc-1.0 database. Distribution (blue), score of possible N-glycosylation sites (e) and positions (f) in amino acid sequences. **(g)** LC-MS/MS-based identification of N-glycopeptide at the N259 site of AZGP1. The LC-MS profiles are shown as spectra averaged over a period of elution time when a representative subset of glycoforms were detected. The cartoon symbols used for the glycans (see inset) conform to the standard representation recommended by the Consortium for Functional Glycomics. Black spot, glycosylated AZGP1; arrowhead, non-glycosylated AZGP1.

**Supplementary Figure 4.** MIIP did not influence the expression of catalytic subunits of oligosaccharide transferase. **(a)** RNA-seq analyses were performed in triplicate on HCT116-MIIP^+/-^ and wild type (MIIP^+/+^) cells, and the datasets were analyzed by GSEA. N-Glycan Biosynthesis GSEA (hallmark gene sets) of the RNA-seq dataset is shown (3 biological replicates per group). **(b)** Heatmap of expression changes in a portion of N-Glycan Biosynthesis-related genes determined from GSEA analysis (3 biological replicates per group). **(c-e)** The mRNA levels of *STT3A* (*Stt3a* in mouse), *STT3B* (*Stt3b* in mouse) in *MIIP* (*Miip* in mouse) stable over-expressing or knock-down HCT116 (c), CT26.WT (d) and CMT93 cells (e) was determined by RT-qPCR (6 biological replicates per group). **(f)** Co-immunoprecipitation analysis of MIIP-AZGP1-STT3A/STT3B interaction in HepG2 cells co-transfected with pCMV-MIIP-Flag and pEX-3-AZGP1-6×His. The lysates were precipitated with anti-His and immunoblot with anti-AZGP1, anti-MIIP, anti-STT3A and anti-STT3B. All data are shown as mean ± SD. ^*^*P* < 0.05, ^**^*P* < 0.01.

**Supplementary Figure 5.** AZGP1 causes WAT browning through β_3_-AR-cAMP-PKA pathway. **(a)** Representative images and statistical analysis of Oil Red O staining in mature adipocytes incubated with the CM from HCT116 (MIIP^+/-^ or WT) cells for 24 h, upon SR59230A (1μM) or DMSO treatment (Scale bar: 50 μm, 3 biological replicates per group). All data are shown as mean ± SD. ^*^*P* < 0.05, ^**^*P* < 0.01, ^***^*P* < 0.001. **(b)** Immunoblots of mitochondrial proteins (UCP1, PGC-1α) and phospho-PKA substrate in mature adipocytes treatment with indicated medium for 24 h, upon SR59230A (1μM), H89-2HCl (20μM) or DMSO treatment. (Representative of 3 biological replicates per group). **(c)** Immunoblots of key proteins in pathways related to inflammation, proliferation, and apoptosis in mature adipocytes treatment with indicated medium for 24 h. (SR59230A: 1μM. Representative of 3 biological replicates per group).

**Supplementary Figure 6.** Murine colorectal cancer cells with Miip reduction aggravates the browning of tumor-adjacent adipocytes in tumor-bearing mouse model. **(a)** Schematic diagram of subcutaneous co-injection model of colorectal cancer cells and differentiated adipocytes in mice. **(b-c)** Stable *Miip* knockdown or scramble CMT93 cells (2 × 10^5^ cells per mouse) were co-injected unilaterally with 3T3-L1 (5 × 10^4^ cells per mouse) into the back of C57B/L6 mice. Tumor growth was monitored (b), and the tumors were removed (c, scale bar: 1cm) at the end of the experiment (n = 5). **(d)** Representative bioluminescence images of tumor growth in mice described in (b) at day 19 (n = 5 mice per group). **(e)** The tumors described in (c) were weighed at the end of the experiment. **(f)** Representative images of H&E staining and IHC staining of UCP1 proteins in allograft tumors derived from (c) (n = 5. Scale bar: 200 μm, black/50 μm, red). All data are shown as mean ± SD. ^*^*P* < 0.05, ^**^*P* < 0.01.

**Supplementary Figure 7.** Free fatty acids are the main fuel for CRC proliferation and survival. **(a)** Parental CMT93 cells treated with two-step CM as described in (Fig.5a), then cell viability was measured with CCK-8 (7 biological replicates per group). **(b-c)** Parental CMT93 (b) or HT29 (c) cells treated with two-step CM as described in (Fig.5a), then cell viability was measured with CCK-8, 7ACC1 (10μM) or SSO (20μM) were added at indicated time point, respectively (7 biological replicates per group). **(d-e)** Parental CMT93 cells treated with two-step CM as described in (Fig.5a), then cell apoptosis induced by oxaliplatin was determined by PI-Annexin V staining (d) followed by flow cytometric analysis and quantification (e), 7ACC1 (5 μM) or SSO (10 μM), (5 biological replicates per group). **(f-g)** Parental HCT116 cells treated with two-step CM as described in (Fig.5a) for 24 h followed by Oil Red O staining, representative images (f) and the quantification of the percentage of average number of Oil Red positive cells (g) were exhibited (Scale bar: 100 μm. 3 biological replicates per group). Adi or Adipo: mature adipocytes. All data are shown as mean ± SD. ^*^*P* < 0.05, ^**^*P* < 0.01, ^***^*P* < 0.001.
